# Comprehensive enhancement of PVC nanocomposites through Al_2_O_3_ for advanced optoelectronics

**DOI:** 10.1038/s41598-025-32078-8

**Published:** 2026-02-05

**Authors:** M. A. Attallah

**Affiliations:** Physics Department, Faculty of Science, Benha University, Benha, 13518 Egypt

**Keywords:** PVC, Al_2_O_3_, Transmittance, Energy loss, Dielectric constant, Optoelectronic applications, Materials science, Nanoscience and technology, Optics and photonics, Physics

## Abstract

With the rapid advancement of elastic and translucent optoelectronics, the demand for low-cost materials has emerged as a major research topic. As a result, this paper investigated how Al_2_O_3_ incorporation altered the properties of PVC. New PVC/xAl_2_O_3_ (x = 0, 0.01, 0.03, 0.07, 0.1, 0.2 wt%) nanocomposites were manufactured by using a cost-effective and simple process (casting method) to adjust the Al_2_O_3_ concentration. The PVC/xAl_2_O_3_ (x = 0, 0.01, 0.03, 0.07, 0.1, 0.2 wt%) nanocomposites were analyzed using X-ray diffraction (XRD), Fourier Transform Infrared spectroscopy (FTIR), scanning electron microscopy (SEM), ultraviolet-visible (UV–visible) spectroscopy, and impedance measurements. Dislocation density (δ), Distortion parameter (g), nanoparticle size (D), and lattice strain (ε) were assessed using the Scherrer and Williamson–Hall methods. Crystal size and the number of crystallites were observed to increase with Al_2_O_3_ content, revealing the higher crystallinity in the material. It was found that the particle size was ~ 30.22 nm for PVC/xAl_2_O_3_ (x = 0.1wt%). SEM analysis showed a consistent distribution of Al_2_O_3_ within the PVC at low concentrations of Al_2_O_3_. These PVC/Al_2_O_3_ films were used as adjustable light- diffusing films in the packaging of different flexible photoelectric devices, according to their visible absorbance characteristic depending on filler concentrations. The optical bandgaps of PVC and PVC/x(Al_2_O_3_) (where x = 0.2) were 5.05 eV and 3 eV, respectively. This decrease was associated with the creation of localized states in the bandgap. The refractive index values obtained were greater than those found in earlier studies, suggesting that the incorporation of a small amount of Al_2_O_3_ nanoparticles improved the refractive index of PVC. It was noted that as the concentration of Al_2_O_3_ rose, the dispersion parameters E_d_, M_2,_ and M_3_ showed an increase, while E_0_ exhibited a decrease. Conversely, the dielectric characteristics of the synthesized nanocomposites improved as the alumina content in the PVC matrix increased. The findings concluded that inexpensive PVC/xAl_2_O_3_(x = 0.1wt%) nanocomposites can be used as an essential component in sophisticated optoelectronic applications.

## Introduction

Developments in materials science and nanotechnology are focused on the creation of inexpensive, flexible, and lightweight materials to meet the needs of contemporary optoelectronic systems^1^. Polymer nanocomposite materials possess distinct optical, electrical, and mechanical characteristics that make them highly desirable from a technological standpoint^2^. These materials are used in a variety of applications, including photovoltaic cells, optical waveguides, magneto-optic data devices, glass lenses, optoelectronic systems, camera lenses, and reflective materials^3–7^.

Organic materials, especially polymers, offer several advantages, including good mechanical properties, low weight, transparency, ease of processing, and cost-effectiveness. However, their refractive index is often relatively low^8^. To adjust and improve polymer optical properties and customize optical variables, such as the refractive index and optical band gap, for optical industrial applications, various nanoparticles, such as Fe_2_O_3_^9^, NaI^10^, graphene oxide^11^, silica^12,13^, copper^14^, iron^15^, and metal oxides^16,17^, NaYF_4_:Eu^+ 3^^18^, have been employed as fillers in various polymer matrix structures.

Specifically, polyvinyl chloride (PVC) is the third most extensively used synthetic polymer that obtained its relevance because of its remarkable features, namely semi-crystallinity, high dielectric constant, high transparency, high elasticity, high absorption in the UV region, low cost, ease of preparation, and non-toxicity^19,20^. Furthermore, its characteristics can be easily enhanced by introducing nanomaterials, metal oxides, and dyes to its matrix. The notable compatibility between PVC and most fillers stems from the ease of bond formation due to the presence of a (-Cl) group in its structure^21^. This enhanced its work in the area of optoelectronic applications^22^. Amongst these fillers, Aluminum oxide, Al_2_O_3_, has been selected since it has excellent dielectric and catalytic capabilities, making it ideal for improving the physicochemical qualities of PVC polymer^23^. Moreover, Al_2_O_3_ is recognized as the most hopeful ceramic material for mounting different electronic devices, plus metal oxide/semiconductor transistors, non-volatile memory cells, etc. Al_2_O_3_ has a significantly higher energy band gap than polymer matrices, making it a promising material for electronic devices^24^.

Based on existing literature, the majority of studies focus on polymer nanocomposites that contain inorganic nanoparticles. Let’s say, Amina et al. examined how different concentrations of ZrO_2_ nanoparticles (NPs) (0, 0.5, 1.5, and 3 weight%) affect the PVC nanocomposite films’ structural properties, dielectric constants, and linear-nonlinear optical features^25^. Doaa et al. created PCL/PVC composites with different weight ratios of CoCl_2_ (0, 2, 4, 8, and 16 wt%). It found that by increasing the weight% of the CoCl_2_ filler, optical band gap values were significantly reduced^26^. Mohamed et al. prepared a PVC/CNT/ZnO nanocomposite for optoelectronic applications. It was found that introducing ZnO reduced the energy gap from 5.4 eV for pure PVC to 4.6 eV for PVC/CNT/ZnO, suggesting a rise in absorption in the visible range^27^. El-Nagger et al. synthesized PVC/ZCO/CdS/PANI/TMAI. It was concluded that the ZCO/CdS/PANI/10 weight% TMAI polymer exhibits a reduction in direct band gap from 4.94 to 3.96 eV^28^. Al-Muntser prepared PVC/PMMA/TiO_2_/GNP for optoelectronic applications. It was found that a 25% rise in the refractive index, which indicates that there are an improvement in photon absorption interactions^29^. Elbasiony et al. synthesized Cu-doped PVC/PE nanocomposites via melt extrusion and investigated their optical and structural properties. Optical studies revealed that light absorption, dielectric response, refractive index, and band gap can be tuned by varying the copper content^30^. Mohamed et al. incorporated graphene nanoplates or multi-wall carbon nanotubes into polyvinyl chloride to enhance the structure and optical performance. It was found that the computed optical parameters varied based on GNPs or MWCNTs^31^. Mohammed et al. used ONCDs to considerably reduce the optical band gap of PVC polymer^32^. BiVO_4_ was found by Yousef et al. to improve the optical polymer properties by shifting the absorption edge from UV to visible and reducing the direct and indirect band gaps from 4.9 to 2.18 eV and 5.17 to 3.04 eV, respectively, when added to the PVP/PVC matrix^33^. Using co-precipitation methods, El-Nagger synthesized polyvinyl chloride (PVC)/(1–x)ZnMn₂O₄/xPbS polymers with and without multi-walled carbon nanotubes (MWCNTs). The effects of the nanofillers on the optical absorbance, reflectance, transmittance, extinction coefficient, refractive index, and energy loss function of the PVC polymer were examined both in the presence and absence of MWCNTs. Overall, the doped PVC exhibited an increase in absorbance and a decrease in transmittance. The direct and indirect optical band gap energies (Eg) of PVC (4.3 and 4.09 eV) decreased after loading with x = 0.1 nanofiller/MWCNTs, reaching minimum values of 3.6 and 2.32 eV, respectively^34^.

While significant changes have been implemented to enhance the performance of PVC, further developments are still necessary to improve its properties for new optoelectronic applications. In this regard, integrating metal oxide nanoparticles like Al₂O₃ is viewed as a promising strategy to strengthen the polymer matrix, increase optical clarity, and adjust its electrical and dielectric characteristics. Consequently, this research centers on the fabrication of PVC/xAl₂O₃ (x = 0, 0.01, 0.03, 0.07, 0.1, 0.2 wt%) nanocomposites through a straightforward and economical casting technique, followed by comprehensive structural, optical, and dielectric analyses. The main goal is to explore how different concentrations of Al₂O₃ affect the optoelectronic performance of PVC, offering insights into its applicability in areas such as flexible sensors, optical devices, and lightweight electronic components. The appropriateness of the derived composites for optoelectronic use is assessed via absorbance measurements within the 200–1100 nm wavelength spectrum. Optical parameters—including refractive index (n), extinction coefficient (k), optical conductivity (σ_opt_), surface energy loss function (SELF), volume energy loss function (VELF), and dissipation factor (tan δ)—are evaluated from both absorption and reflection spectra. Furthermore, the optical band gap is derived from the absorption coefficient, and the dielectric characteristics are analyzed. The originality of this research resides in creating links between structure and optical properties, showcasing the potential of Al₂O₃ nanoparticles to collectively enhance the performance of PVC, thus offering a cost-effective route to advanced polymer-based optoelectronic materials.

## Experimental

### Materials

Polyvinyl chloride (PVC) powder, with an approximate average molecular weight of Mw ≈ 233.000, was acquired from Sigma-Aldrich and is of a high-purity grade suitable for optical and electrical investigations. Aluminum oxide (Al₂O₃) nanoparticles, used as the reinforcing filler, were obtained from NanoTech Egypt; 30–50 nm; ≥99% purity. All materials were employed without any further purification. Tetrahydrofuran (THF, with a purity of ≥ 99.5%), of analytical grade, was used as the solvent and sourced from Sigma-Aldrich.

### PVC/Al_2_O_3_ films preparation

Nanocomposite films were created utilizing a solvent-casting method. Initially, polyvinyl chloride (PVC) powder (Sigma-Aldrich) was mixed with tetrahydrofuran (THF; Purity, ≥ 99.5%) and stirred magnetically at room temperature (25 ± 2 °C) for 1 h until a clear and uniform solution was formed. Aluminum oxide nanoparticles (Al₂O₃, NanoTech Egypt; 30–50 nm; ≥99% purity) were introduced into the PVC solution at concentrations of 0.01, 0.03, 0.07, 0.10, and 0.20 wt%. To achieve uniform distribution and prevent agglomeration of the particles, the mixture was stirred continuously for 2 h, followed by sonication in an ultrasonic bath at 40 kHz for 30 min. The prepared nanocomposite solutions were poured into leveled glass Petri dishes and allowed to dry at 60 °C for 48 h to ensure complete evaporation of the solvent and stabilization of the film. After drying, the films were cooled to room temperature and gently peeled off the substrates. To relieve any residual stresses and enhance interfacial bonding, the samples underwent annealing at 60 °C for an additional hour in a controlled oven environment. The final films exhibited a smooth, crack-free surface and were optically transparent. The thickness of the films was measured using a digital micrometer (with ± 1 μm accuracy) at five random positions on each sample, resulting in an average thickness ranging from 100 to 130 μm depending on the nanoparticle content. All prepared samples were stored in desiccators to prevent moisture absorption before characterization.

### Instruments

A Rigaku Miniflex 600 diffractometer with Cu Kα radiation, λ = 1.5418Å, was used to analyze the materials’ structure using the X-ray diffraction method. An FTIR spectrophotometer (Model: Nicolet DTGS TEC detector) was used to detect the Fourier Transform Infrared (FTIR) spectra of prepared crystals in a wavenumber range between 400 and 4000 cm^− 1^. Quanta FEG 250 was used to study the surface morphology of the prepared samples. A UV–VIS spectrophotometer of the Edinburgh DS5 dual beam spectrophotometer was used to measure the optical characteristics in the wavelength range of 200–1100 nm. Dielectric characteristics in the frequency range of 100 Hz to 10 MHz were investigated using the RLC − 8110 G bridge.

## Results and discussions

**Fig. 1:**
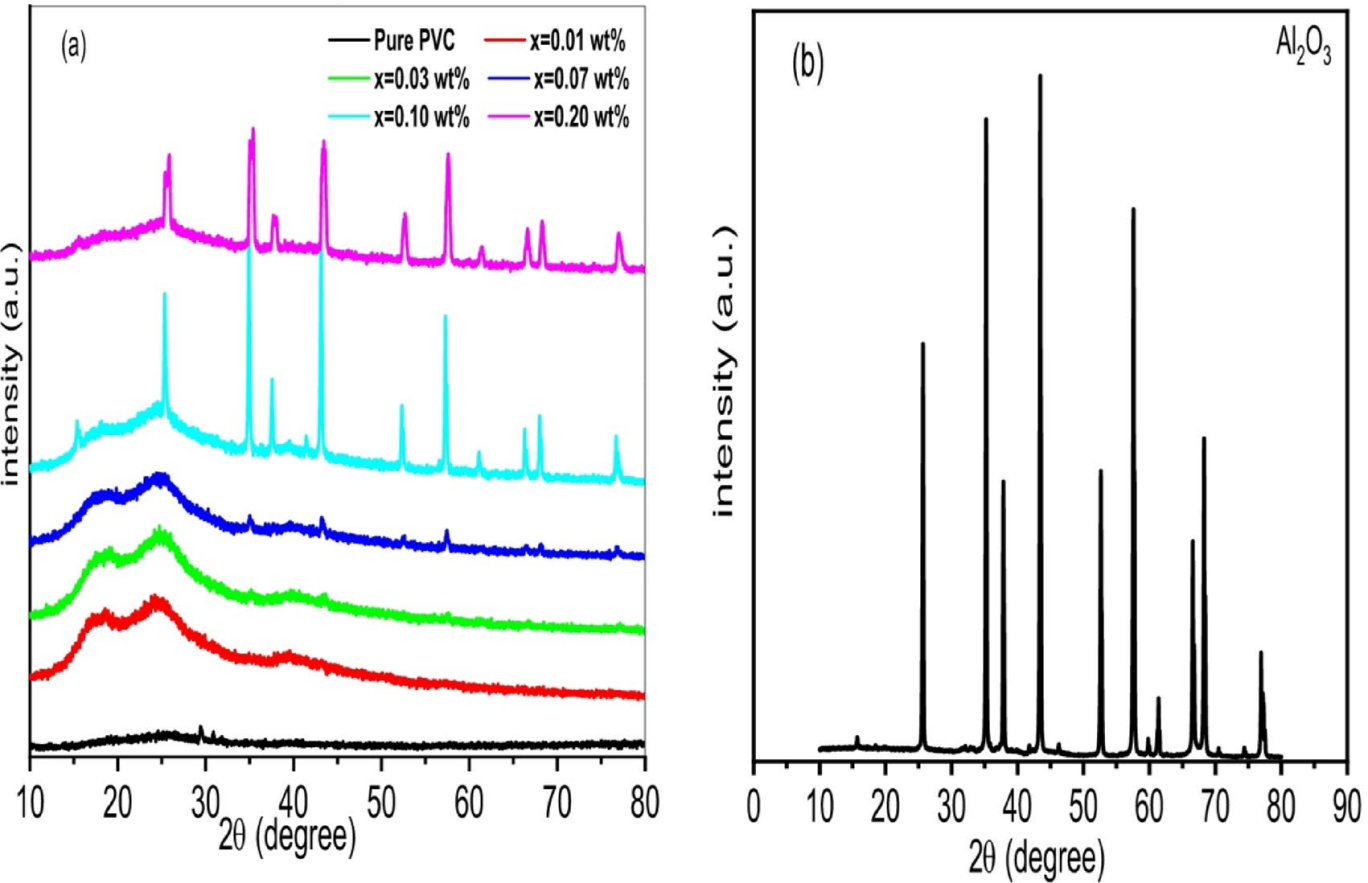
**(a)** XRD of PVC/x(Al_2_O_3_) films (x = 0, 0.01, 0.03, 0.07, 0.1, 0.2 wt%) **(b)** XRD of Al_2_O_3_.

**Fig. 2:**
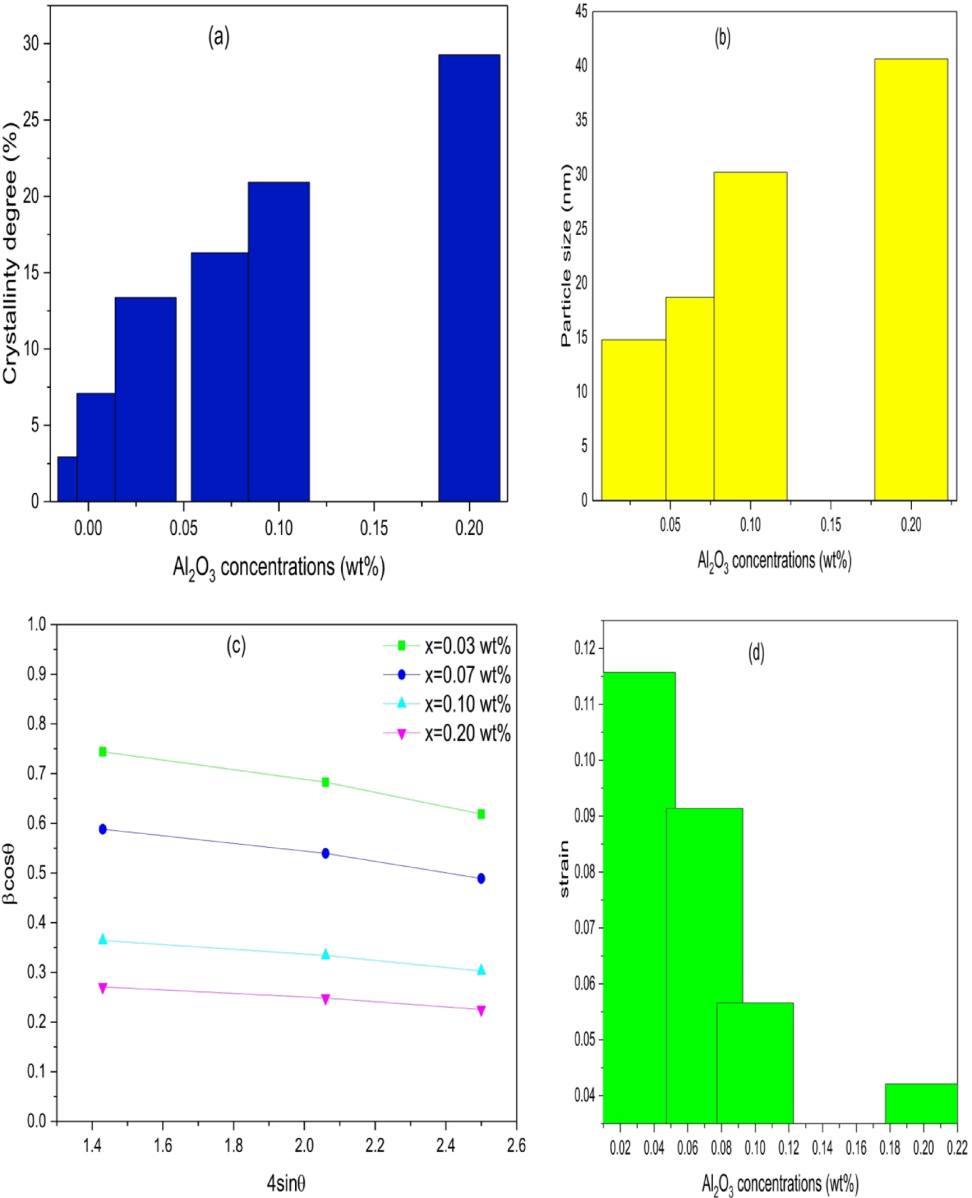
**(a)** Variation of crystallinity degree with Al_2_O_3_ concentrations. **(b)** Variation of particle size with Al_2_O_3_ concentrations. **(c)** plot of β cos θ against 4sin θ for PVC/x(Al_2_O_3_) nanocomposites (x = 0.03, 0.07, 0.1, 0.2 wt%) **(d)** Variations of strain with Al_2_O_3_ concentrations.

Figure 1a. demonstrates the X-ray patterns for PVC/x(Al_2_O_3_) films (x = 0, 0.01, 0.03, 0.07, 0.1, 0.2 wt%). A wide peak in the 2θ range (17°–28°)^35^ indicates the pure PVC film amorphicity^20^. Nonetheless, the tiny peak at 29° suggests that PVC may have an ordered structure^36^. By introducing Al_2_O_3_ into the PVC matrix, the PVC main peak intensity increases, and its broadening decreases. Furthermore, distinct peaks are detected at 2θ = 35, 38, 42, 52, 56, 67, 68 and 78° which are interrelated to (104), (110), (006), (024), (116), (214), (300) and (119) planes of crystalline Al_2_O_3_ with lattice parameters (a = b = c = 0.47 nm) according to DB Card Number (10–0017) which was confirmed by measuring the X-ray pattern of Al_2_O_3_ as in Fig. 1b. This peak’s intensity also increases with Al_2_O_3_ content, indicating a growth in the crystalline structure of the composite samples. Consequently, the relation (Xc=$\:\frac{A}{{A}_{0}}$*100)^37^ is used to determine the degree of crystallinity (Xc), where A implies to area under the crystalline peak and A_0_ implies the area under the entire pattern. Figure 2a. shows the variation of crystallinity degree with Al_2_O_3_ contents. Due to changes in the amorphous structure, it is evident that the degree of crystallinity increases as the Al_2_O_3_ concentration increases^38^. The more defined and pronounced XRD peaks are seen at 0.1 and 0.2 wt% concentrations of Al₂O₃ within the PVC matrix indicate a noticeable increase in crystallinity and structural order compared to lower levels, such as 0.01, 0.03, and 0.07 wt%. At these elevated concentrations, Al₂O₃ is likely functioning as a highly effective nucleating agent, encouraging the development of more organized crystalline areas within the polymer^39^. This leads to stronger diffraction signals due to better alignment and packing of the PVC chains. Furthermore, the distribution of Al₂O₃ particles is more consistent at 0.1 and 0.2 wt%, promoting improved interaction with the polymer matrix and aiding in structural reorganization^40^. In contrast, lower concentrations might not generate enough nucleation sites or could result in poor dispersion, leading to broader, less intense peaks that indicate a more amorphous or disordered structure^41^. The composition of the Al₂O₃ phases also has an effect, as higher concentrations may enhance the emergence of more crystalline phases such as α-Al₂O₃, which further contributes to the sharpness of the peaks^42^. In summary, the trend observed underscores the significance of adjusting additive concentration to improve the structural characteristics of PVC composites.

Increased crystallinity causes structural variations, such as grain changes and strain within the crystalline region. To investigate this variance, the average crystallite size (D) is determined by Scherer’s equation (D=(0.9*λ)/βcosθ)^43^ where λ is the wavelength of the X-ray, β is the diffraction peak’s full width at half maximum, and θ is the diffraction Bragg angle. The crystal size, as in Fig. 2b, and the number of crystallites increase with Al_2_O_3_ content, revealing the region’s higher crystallinity. The strain (ε) is calculated using the relation (βcosθ =$\:\:\frac{k\lambda\:}{D}$+4ε sinθ)^44^ as in Fig. 2c. Figure 2d shows that the strain decrease with increasing Al_2_O_3_ content. The reduction in strain as the Al₂O₃ content increases can be explained by the limited mobility of the polymer chains due to the presence of rigid ceramic nanoparticles. Al₂O₃ particles serve as physical crosslinking sites within the PVC chain, restricting the ability of the chains to elongate and deform when stress is applied. Moreover, the strong interactions at the interface between the nanoparticles and the PVC chains bolster the composite’s stiffness, which results in decreased ductility. The gradual occupation of free volume by the nanoparticles further hinders chain slippage, leading to diminished strain values as the Al₂O₃ concentration rises. The dislocations (δ), which are described as the number of dislocation lines per unit of crystal volume, are evaluated through $\:\left(\:{\updelta\:}=\frac{1}{{D}^{2}}\right)$^45^. The distortion parameters (g) are calculated by (g=$\:\frac{\beta\:}{tan\theta\:}$)^46^. Table 1 contains a tabulation of all determined values.

**Table 1:** Crystallinity degree(X_C_), crystal size (D), strain ($\:\varepsilon\:)$, dislocation density ($\:\delta\:$), and distortion parameters (g) for PVC/x(Al_2_O_3_) films (x = 0, 0.01, 0.03, 0.07, 0.1, 0.2 wt%).

Concentrations (wt%)	Crystallinity degree(%)	Crystal size from S_E (D) (nm)	Crystal size from W_H (D) (nm)	Strain ($\:\varepsilon\:\times\:{10}^{-3})$	Dislocation density ($\:\delta\:\times\:{10}^{-3}$) (lines nm^− 3^)	Distortion parameters(g$\:\times\:{10}^{-4}$) (nm)
Pure PVC	2.93	-	-	-	-	-
0.01	7.09	-	-	-	-	-
0.03	13.37	17.12	14.79	115.72	45.72	42.5
0.07	16.30	21.47	18.71	91.4	28.57	33.6
0.10	20.92	34.69	30.22	56.6	10.95	20.8
0.20	29.27	46.66	40.64	42.1	6.05	15.5

**Fig. 3:**
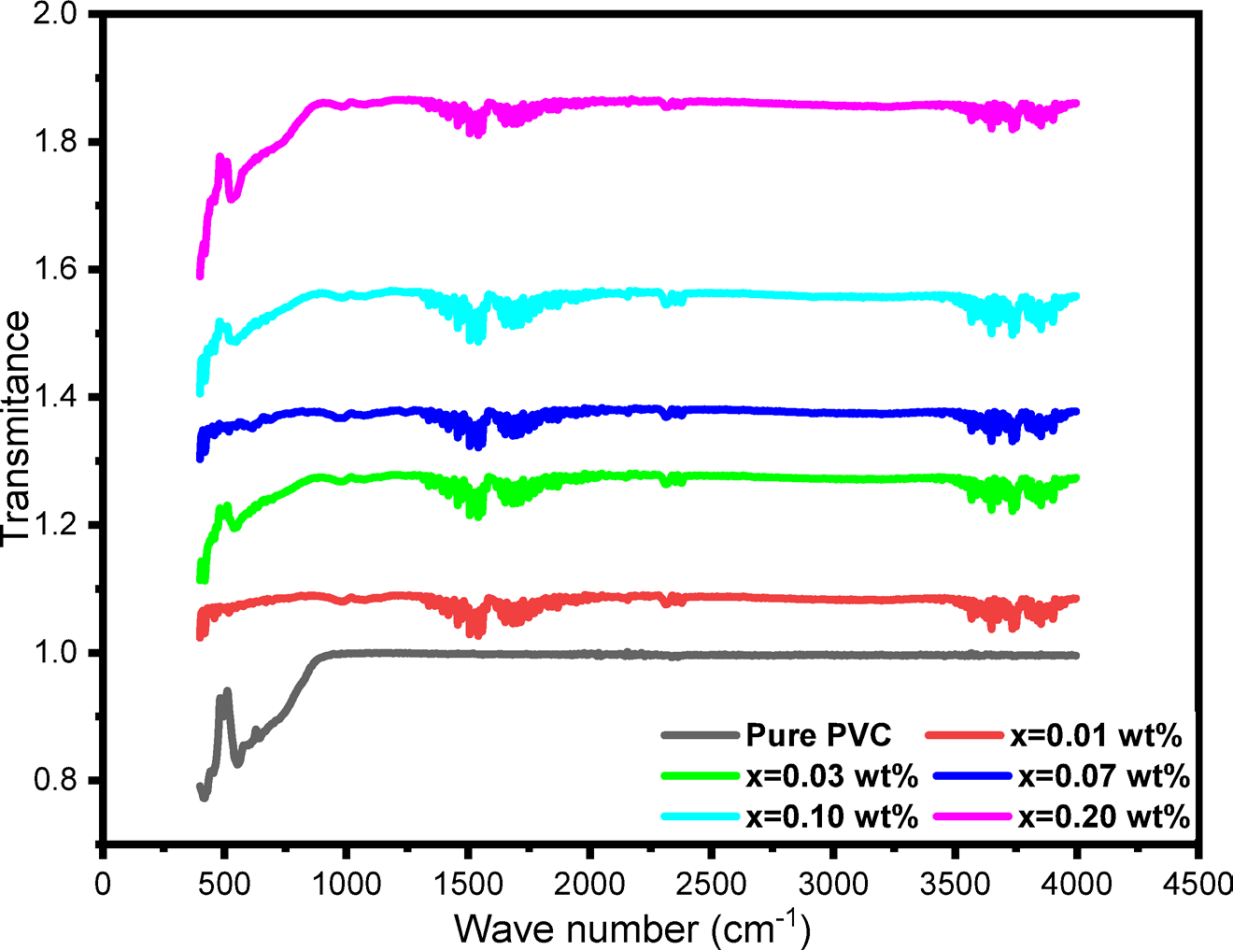
FTIR of PVC/x(Al_2_O_3_) films (x = 0, 0.01, 0.03, 0.07, 0.1, 0.2 wt%).

Figure 3. shows the FTIR spectra for both pure PVC and PVC/xAl₂O₃ (x = 0, 0.01, 0.03, 0.07, 0.1, 0.2 wt%) composites. The distinctive bands of pure PVC are prominently visible, including the C–Cl stretching vibration found in the range of 600–700 cm⁻¹^47,48^, along with the C–H bending of CH₂ groups, which appears around 1420–1450 cm⁻¹^47^. The C–H stretching region between 2850 and 3000 cm⁻¹ is also clearly defined, affirming the typical functional vibrations related to the PVC backbone^49^. The introduction of Al₂O₃ nanoparticles does not introduce any new absorption peaks, suggesting that there are no chemical reactions or structural damage to the PVC chains^40^. Nevertheless, significant changes in the intensity and transmittance of the primary absorption bands are observed with increased filler concentrations. These spectral changes indicate the formation of interfacial interactions between the Al₂O₃ nanoparticles and the polar C–Cl dipoles in the PVC matrix^50^. A wide band appears in the high-wavenumber range (3500–4000 cm⁻¹), which is absent in pure PVC is associated with the O–H stretching of hydroxyl groups on the surface of Al₂O₃ nanoparticles and/or adsorbed water molecules^51^. Its gradual increase with the concentration of nanoparticles confirms the incorporation of Al₂O₃ in the polymer matrix and indicates hydrogen-bond-like interfacial interactions with the PVC chains, which enhance structural cohesion^52^. Such interactions are anticipated to limit chain mobility and, in turn, decrease free volume, which leads to improved ordering of the polymer chains around the nanoparticles^53^. This phenomenon illustrates a type of physical cross-linking, which results in enhanced polymer arrangement and greater structural stability^54^. In summary, the FTIR findings confirm that the nanocomposites maintain the chemical integrity of PVC, while the presence of Al₂O₃ enhances interfacial compatibility, potentially benefiting the improvement of the material’s optical properties.

**Fig. 4:**
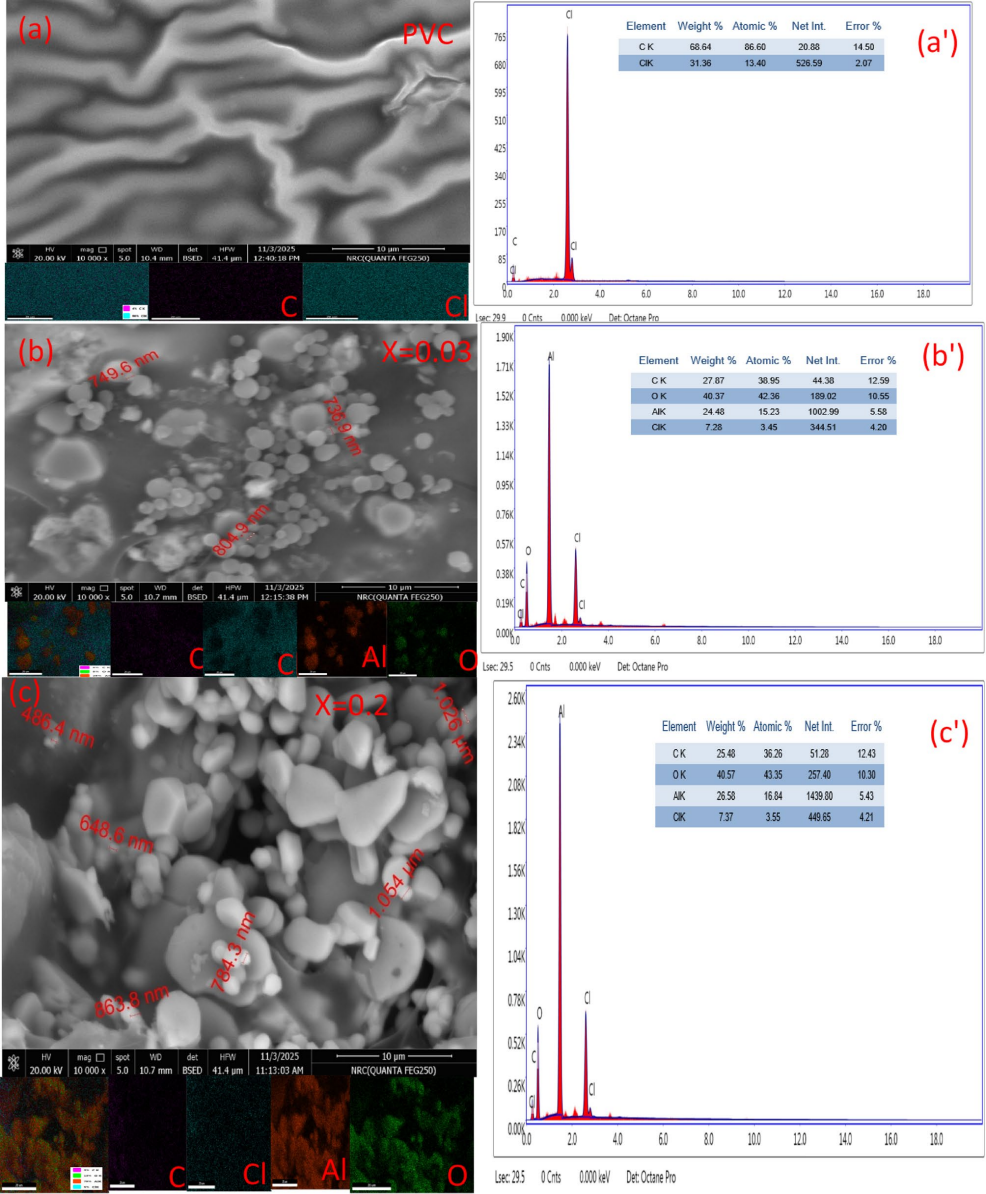
SEM images, EDX spectra, and elemental mapping of PVC/xAl₂O₃ nanocomposites (x = 0, 0.03, 0.2 wt%).

The surface characteristics and elemental makeup of the synthesized PVC/xAl₂O₃ (x = 0, 0.03, 0.2 wt%) nanocomposites are analyzed using SEM-EDX, as shown in Fig. 4. The SEM image of pure PVC (Fig. 4(a)) displays a smooth and layered surface, typical of its largely amorphous nature^55^. No noticeable particulate features can be seen, indicating the absence of any foreign phases. This finding is corroborated by the corresponding EDX spectrum (Fig. 4(a′)), which reveals only the peaks for C and Cl at the expected elemental ratios, affirming the purity of the PVC matrix. With the addition of a low concentration of Al₂O₃ nanoparticles (0.03 wt%), a significant change in surface texture is observed (Fig. 4(b)). The morphology shifts from a smooth layered finish to a rougher and more granular structure, signifying the successful integration of nanoparticles into the polymer^56^. The particles appear to be well-dispersed, forming nanoscale clusters without considerable agglomeration. Such dispersion implies a strong interaction between the Al₂O₃ particles and the PVC chains^57^. The accompanying EDX spectrum (Fig. 4(b′)) shows the emergence of Al and a noticeable rise in the intensity of the O signal, confirming the presence of Al₂O₃. Elemental mapping further illustrates a uniform distribution of C, Cl, Al, and O across the surface, validating the effective incorporation of the nanofiller and uniform dispersion. At higher Al₂O₃ concentrations (0.2 wt%) (Fig. 4(c)), the polymer surface takes on a more irregular appearance, exhibiting dense clusters and larger aggregates, with particle sizes extending into the sub-micron range. This morphology indicates particle agglomeration, which is typically linked to an increase in nanoparticle concentration and restricted polymer chain movement around compacted fillers^58^. EDX analysis (Fig. 4(c′)) shows an increased intensity of Al and O signals, along with a corresponding reduction in C and Cl contributions, consistent with higher Al₂O₃ content. Elemental mapping confirms the presence of all the constituent elements, though localized clusters suggest partial agglomeration at higher loadings. In summary, SEM-EDX analysis verifies the successful incorporation of Al₂O₃ nanoparticles into the PVC matrix, showcasing homogeneous dispersion at lower concentrations and observable agglomeration at higher filler levels. The gradual alterations in morphology and elemental composition are directly related to the increasing amount of nanofiller, demonstrating effective nanocomposite formation and robust interactions between the polymer and filler.

**Fig. 5:**
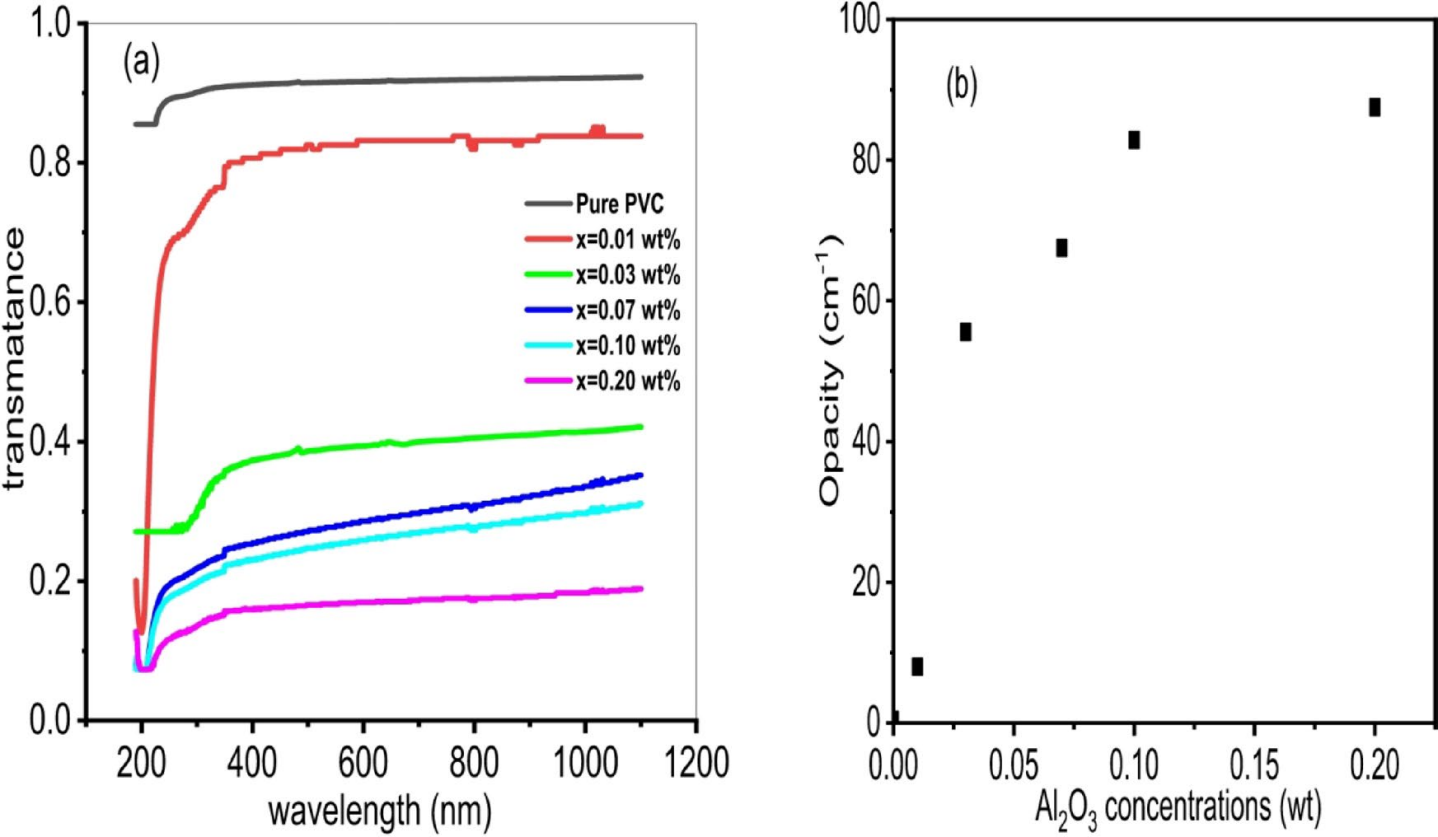
**(a)** transmission spectrum of PVC/x(Al_2_O_3_) composite (x = 0, 0.01, 0.03, 0.07, 0.1, 0.2 wt%) **(b)** Dependence of Opacity on Al_2_O_3_ concentration.

Figure 5a. shows the transmittance spectrum of the PVC/x(Al_2_O_3_) composite (x = 0, 0.01, 0.03, 0.07, 0.1, 0.2 wt%). In the UV region (200–400 nm), transmittance increases with increasing wavelength. This increase is related to the PVC band gap^59^. In the visible region (400–1100 nm), transmittance changes very slowly with the wavelength. This is due to the normal dispersion of light^60^. It is evident that when the concentration of Al_2_O_3_ nanoparticles rises, the transmission falls. This results from the agglomeration altering the size of the Al_2_O_3_ nanoparticles in the PVC matrix^61^. At around 400 nm, the optical transmission maximum (nearly 90%) for pure PVC has been determined. For the nanocomposite that contains 0.2 weight% Al_2_O_3_ nanoparticles, this value decreases to about 12%. The significant morphological changes seen in the SEM images correspond with the notable decrease in transmittance (from approximately 90% to around 12%). The change from a smooth PVC surface to densely aggregated Al₂O₃ particles, particularly at increased loadings, creates notable light-scattering sites and amplifies interfacial variation^62^. These structural characteristics promote photon scattering and shorten optical pathways, which accounts for the considerable reduction in transparency^63^. This relationship indicates that the agglomeration of nanoparticles, rather than just the addition of filler, is the primary factor in the sharp decline in transmittance^64^. Opacity was studied to verify the transparency of the film since transparency decreases with increasing opacity^65^. Figure 5b. shows the opacity of the PVC/x(Al_2_O_3_) composite (x = 0, 0.01, 0.03, 0.07, 0.1, 0.2 wt%). Since PVC is a transparent material and SEM analysis shows that nanoparticle aggregation inside the polymer matrix may be the cause of the decrease in PVC transparency. It was found that as the concentration of Al_2_O_3_ rises, so does the opacity. This increase is due to light- scattering effects^66^.

**Fig. 6:**
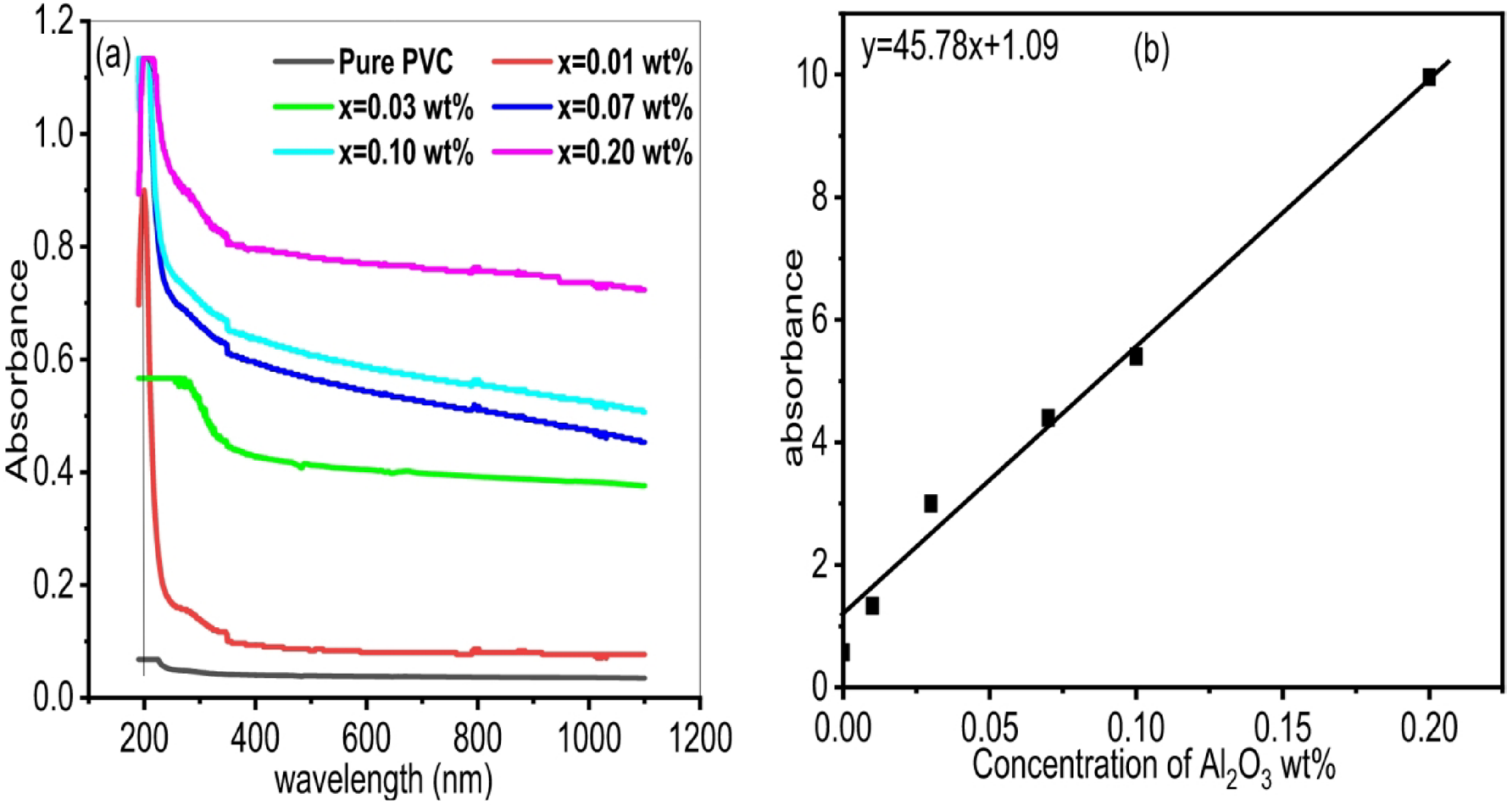
**(a)** Absorption spectrum of PVC/x(Al_2_O_3_) composite (x 0, 0.01, 0.03, 0.07, 0.1, 0.2 wt%) **(b)** Dependence of absorbance on Al_2_O_3_ concentration.

Figure 6a. shows the absorbance spectrum of PVC/x(Al_2_O_3_) composite (x = 0, 0.01, 0.03, 0.07, 0.1, 0.2 wt%). There is an absorption edge for pure PVC samples in the UV area at λ = 205 nm. This edge is allocated to the electron migration of the C = C unsaturated bond of the PVC π – π* transition^67^. Furthermore, all polymer films’ absorbance drops off significantly between 200 and 400 nm. The absorbance decreases more slowly in the visible range than in the UV region. It is evident that as the concentration of Al_2_O_3_ nanoparticles rises, the absorbance of the polymer films increases, and the absorption edge moves to higher wavelengths (redshift). This is linked to the aggregation of nanoparticles in the PVC polymer^68^. The absorption edge’s redshift provides evidence of the polymer films’ altered optical band gap. These PVC films may be used as adjustable light-diffusing films in the packaging of different flexible photoelectric devices, according to their visible absorbance characteristic depending on filler concentration. Also, these findings suggested that loaded PVC polymers could be widely used in adhesives and pharmaceuticals^69^. While the sample with 0.20 wt% shows the highest absorbance, the steep increase indicates potential scattering losses and agglomeration of nanoparticles. Therefore, 0.10 wt% Al₂O₃ is identified as the optimal concentration, as it offers considerable optical enhancement while preserving a consistent and stable nanocomposite structure that is ideal for optoelectronic applications. Figure 6b. depicts the variation of absorbance with aluminum oxide concentrations. The absorbance increased with increasing aluminum oxide concentrations. This is because, by functioning, for example an inducer to the PVC medium, the incorporated Al_2_O_3_ nanoparticle creates charge-transfer centers^40^. The correlation between absorbance(A) and aluminum oxide concentrations (C) is found (A = 45.78 C + 1.09 with R^2^ = 0.9859). From this, it’s clear that there is a large degree of agreement between the practical results and the theoretical model.

When evaluating optoelectronic materials, the refractive index (n) and extinction coefficient (k) are thought to be the primary optical properties that must be studied. The formulas below can be used to calculate these parameters^70^:

1 $$\:\mathrm{K}=\frac{\alpha\:\lambda\:}{4\pi\:}$$

2 $$\:\mathrm{n}=\frac{(1+R)}{(1-R)}+\sqrt{\frac{4R}{({1-R)}^{2}}}-{K}^{2}$$

**Fig. 7:**
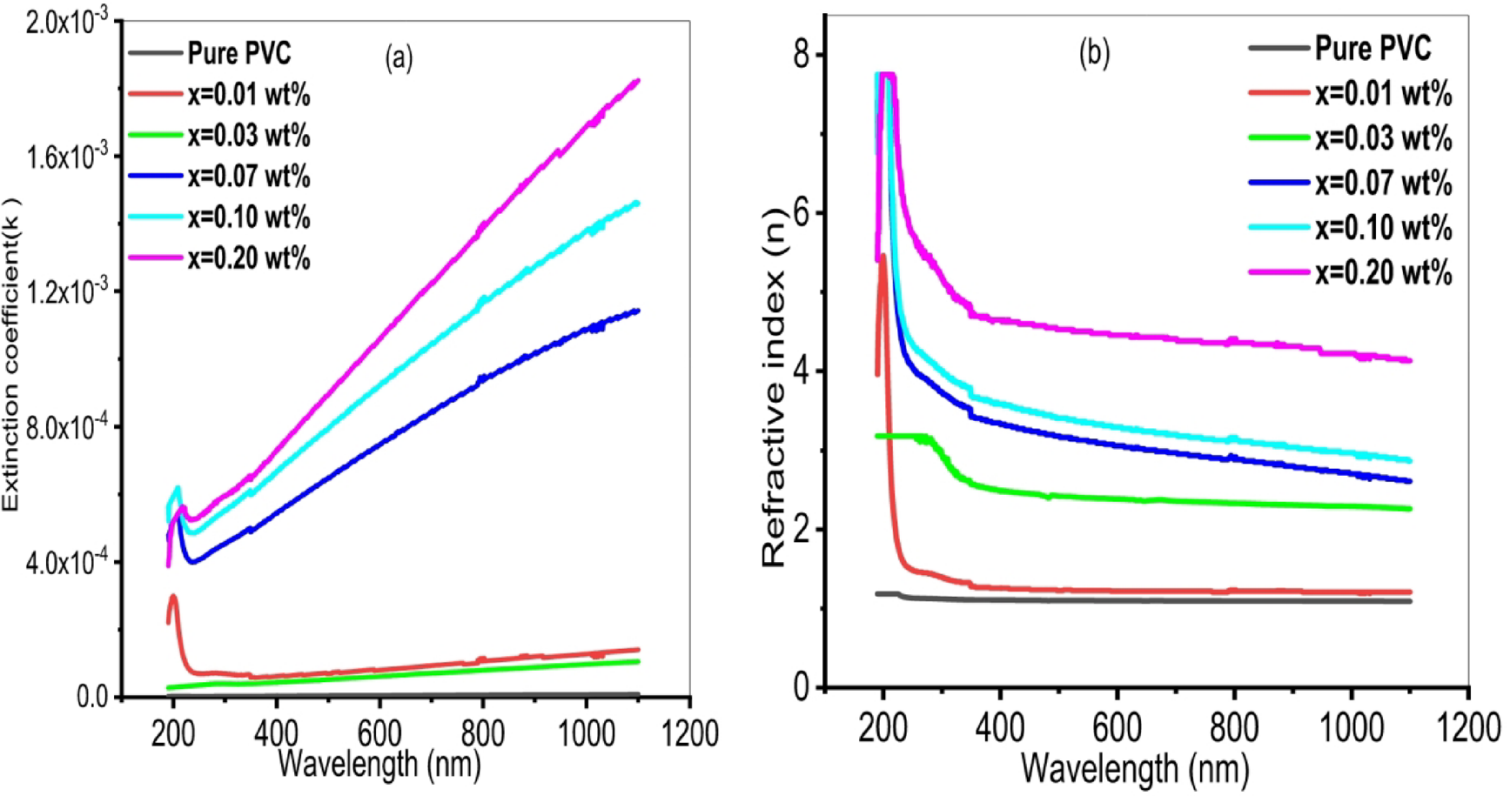
**(a)** Variation of extinction coefficient and **(b)** Refractive index of PVC/x(Al_2_O_3_) composite (x 0, 0.01, 0.03, 0.07, 0.1, 0.2 wt%) with wavelength.

Where R represents film reflectance, $\:\alpha\:$ refers to the film absorption coefficient. Photonic materials are characterized by the extinction coefficient k, which measures the decrease in transmitted light brought on by absorption and scattering. Also, it indicates the likelihood that the electronic transition will occur if it were to be studied. Figure 7a. illustrates the extinction coefficient of PVC/x(Al_2_O_3_) composite (x 0, 0.01, 0.03, 0.07, 0.1, 0.2 wt%). In the low wavelength range (200–250 nm), the extinction coefficient falls. One possible explanation for this could be that the incident photon possesses enough energy to excite the electron from its initial position to a different one. This indicates a drop in energy loss, which results in a decrease in the extinction coefficient. The extinction coefficient also rises dramatically at high wavelengths in the 400–1100 nm range, where the input photon lacks sufficient energy to activate the electrons. Consequently, a significant extinction coefficient resulted from the substantial energy loss. These outcomes concur with the data that was previously seen^71^. It is evident that for every film, the extinction coefficient rises with the concentration of Al_2_O_3_ nanoparticles.

Figure 7b. clarifies the refractive index of PVC/x(Al_2_O_3_) composite (x 0, 0.01, 0.03, 0.07, 0.1, 0.2 wt%). Prepared composites exhibit wavelength-dependent dispersion behavior. The refractive index drops off dramatically as the wavelength increases until λ = 400 nm. While in the visible area, the refractive index practically stays constant between 500 and 1100 nm. As the number of Al_2_O_3_ nanoparticles increases, it is evident that the refractive index (n) rises. For example, at λ = 500 nm, the refractive index of pure PVC rises from 1.2 to 4.9 when 0.2 wt% Al_2_O_3_ nanoparticles are entering into the PVC matrix. This results from aluminum ions creating hydrogen bonds with nearby polymer chains, which raises the density^72^. The n values that are obtained are higher than what was stated in literature^73^, which means that by adding fewer Al_2_O_3_ nanoparticles, the PVC’s refractive index can be adjusted.

Using Tauc’s relation, the energy band gap (E_g_) for each sample is determined by calculating the absorption coefficient (α) and photon energy (hv), as indicated in the following^74^:

3 $$\:\alpha\:\mathrm{h}\nu\:=\mathrm{B}{(\mathrm{h}\nu\:-{E}_{g})}^{n}$$

**Fig. 8:**
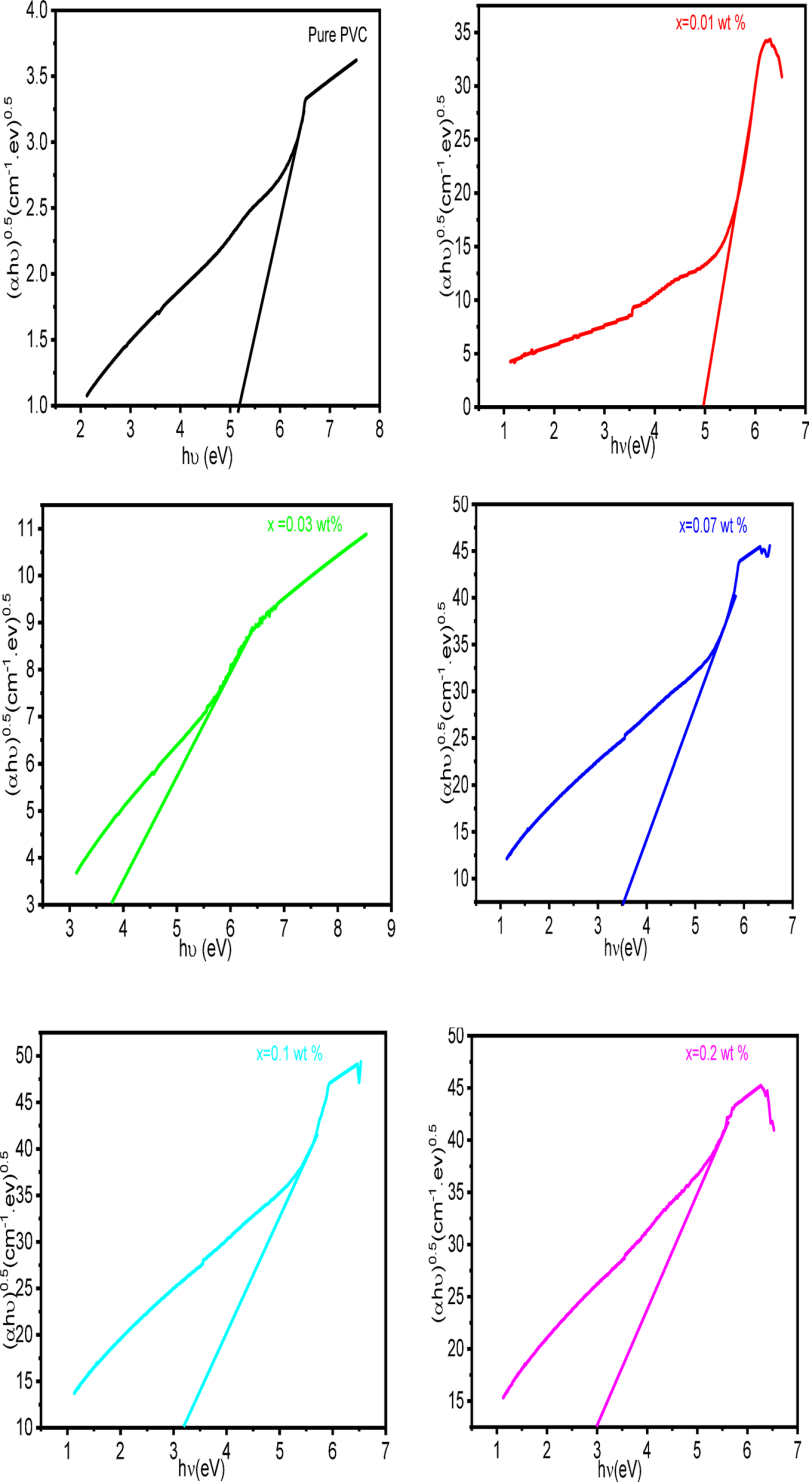
Plot (αhυ)^0.5^ against (hυ) for PVC/x (Al_2_O_3_) composite (x = 0, 0.01, 0.03, 0.07, 0.1, 0.2 wt%).

Where B is constant and n represents the power symbol for the permitted transition (*n* = 0.5 for indirect transitions and *n* = 2 for direct transitions). The indirect optical band gap is obtained by applying the least square fitting approach on the linear portion of the (αhυ)^0.5^ against the (hυ) plot, as in Fig. 8. The calculated indirect energy band gap is recorded in Table 2. The indirect energy band gap decreases with increasing concentrations of Al_2_O_3_. This decrease is associated with the creation of localized states in the band gap^75^. The reduction in band gap with increased loading of Al₂O₃, despite a rise in crystallinity, can be explained by the emergence of localized interfacial states between PVC and Al₂O₃, which serve as intermediate energy levels close to the band edges^76^. Additionally, the increased refractive index and enhanced charge delocalization linked to strong interactions between the nanoparticles and the polymer promote electronic transitions, which in turn reduce the effective optical band gap^77,78^. Therefore, the effects of interfacial electronic interactions outweigh the anticipated increase in band gap associated with enhanced crystallinity^79^.

**Fig. 9:**
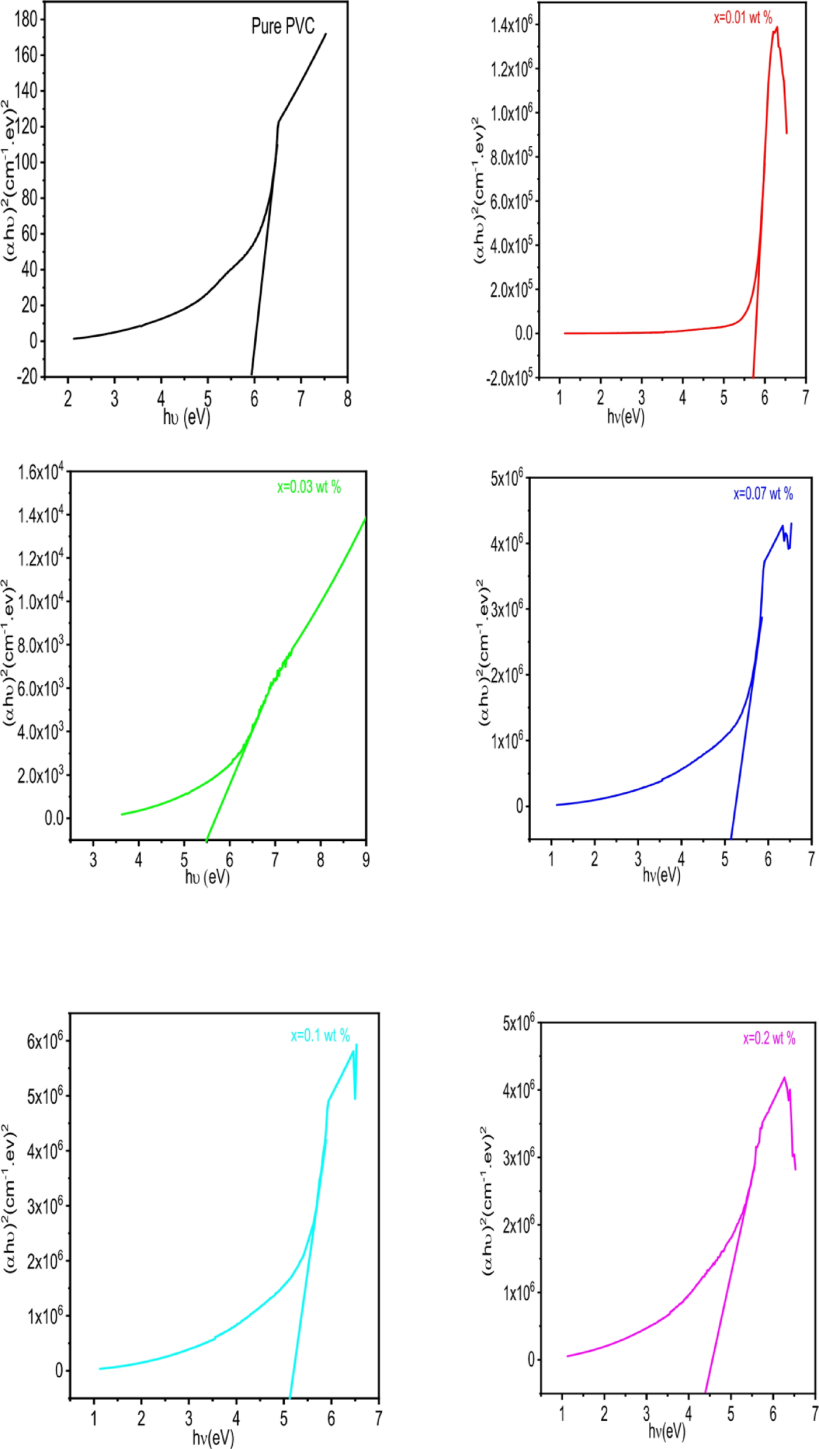
Plot (αhυ)^2^ against (hυ) for PVC/x (Al_2_O_3_) composite (x 0, 0.01, 0.03, 0.07, 0.1, 0.2 wt%).

Also, the linear part of the (αhυ)^2^ against (hυ) plot is used to fit the direct optical band gaps using least squares, as shown in Fig. 9. For the PVC/xAl_2_O_3_ (x = 0, 0.01, 0.03, 0.07, 0.1, 0.2 wt%) composite, the direct energy band gaps are shown in Table 2. It is observed that the optical band gap for pure PVC is 5.9, and for PVC with 0.2 weight% of Al_2_O_3_ nanoparticles is 4.45. This reduction in the direct optical band gap is linked to the formation of localized states in the band gap and serves as a sign of the alteration in the polymer matrix’s structure. The EDX analysis indicates the presence of aluminum and oxygen in the composite, evidenced by strong peaks from both elements, in addition to the anticipated carbon and chlorine signals from PVC. This supports the conclusion that the observed changes in spectral and optical properties stem from embedded Al₂O₃ (or oxidized aluminum species) rather than an impurity or measurement artifact. The strong signals for both oxygen and aluminum align with the existence of Al₂O₃ particles and suggest the presence of surface hydroxyl groups (O–H) indicated by FTIR measurements at approximately 3400 cm⁻¹. Coupled with SEM observations of sub-micron and micrometer-scale particles or aggregates, the EDX results suggest that (i) Al₂O₃ is present but not evenly distributed, leading to localized aluminum-rich areas that enhance light scattering and alter path-length variability, and (ii) that the oxygenated surface species at the interface of Al₂O₃ and PVC may create tail and mid-gap states, promoting increased absorption below the band gap. Consequently, the noticeable reduction in apparent band gap energy is likely attributed to (a) interfacial defect or charge-transfer states (such as oxygen vacancies, hydroxyl groups, and Al–O–polymer connections) that bolster sub-gap absorption^80^, and (b) optical artifacts resulting from particle aggregation and scattering, rather than a uniform reduction in the intrinsic band gap of bulk PVC^81^.

**Table 2:** The optical energy band gap for PVC/x (Al_2_O_3_) composite (x 0, 0.01, 0.03, 0.07, 0.1, 0.2 wt%).

Concentrations (wt%)	Indirect band gap (eV)	Direct band gap (eV)
Pure PVC	5.05	5.9
0.01	5	5.7
0.03	3.7	5.5
0.07	3.5	5.13
0.1	3.2	5.1
0.2	3	4.45

**Fig. 10:**
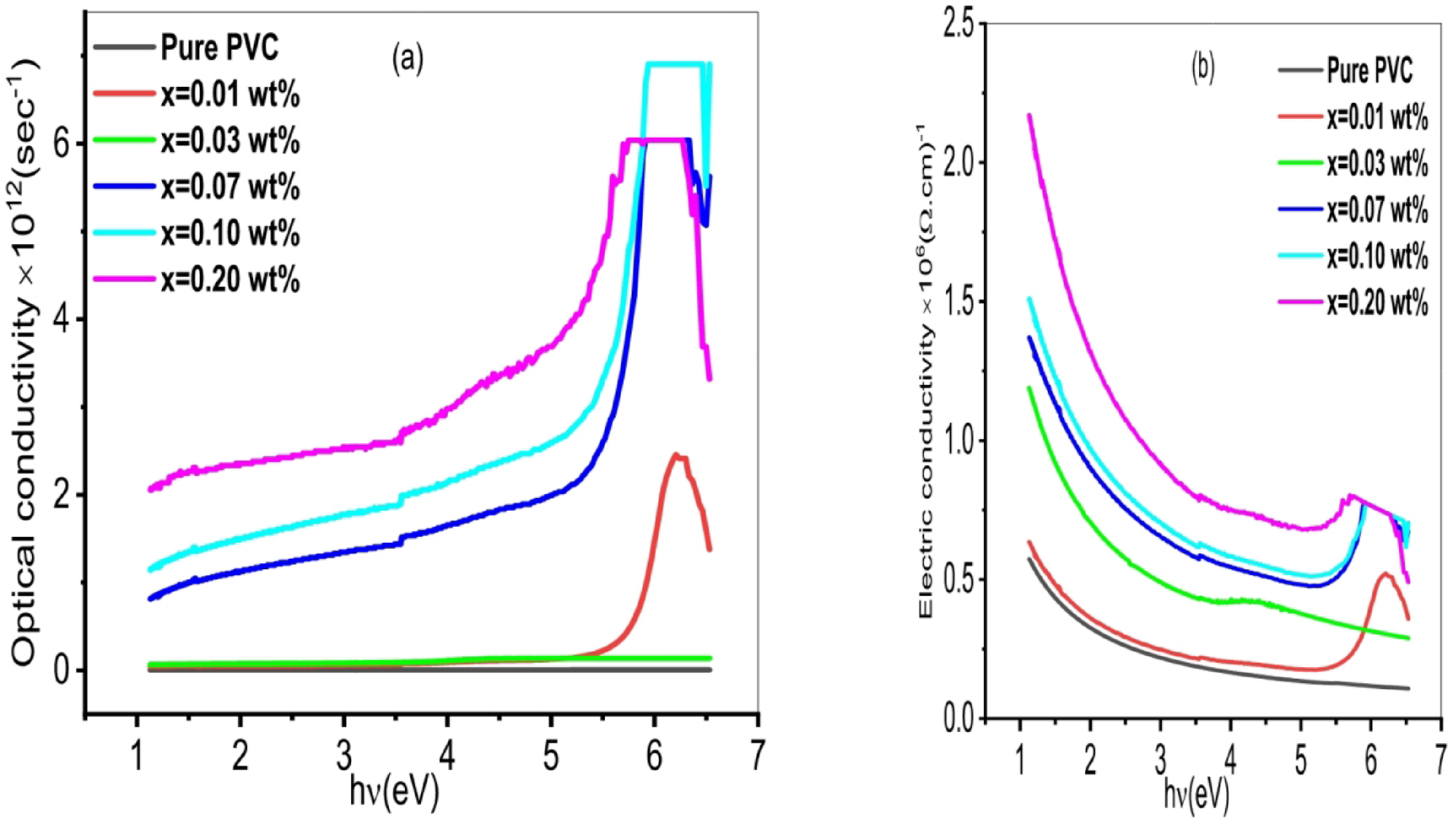
**(a)** Dependence of optical and **(b)** Electrical conductivity of PVC/x (Al_2_O_3_) nanocomposite (x 0, 0.01, 0.03, 0.07, 0.1, 0.2 wt%) on photon energy.

Since the optical conductivity σ_opt_ represents the optical reaction of the material to the diffusion of charge carriers as a result of excitation brought on by the incident photon^14^. The refractive index (n) and absorption coefficient of the materials ($\:\alpha\:)\:$directly affect optical conductivity ($\:{\sigma\:}_{opt})$, as shown by the following equation^38^:

4 $$\:{\sigma\:}_{opt}\:=\frac{n\alpha\:C}{4\pi\:}$$

where C is the vacuum speed of light. Figure 10a. displays the optical conductivity dependences on incident photon energy for PVC/x(Al_2_O_3_) (x = 0, 0.01, 0.03, 0.07, 0.1, 0.2 wt%). Two areas are found, optical conductivity appears to remain constant in the low photon energy area (< 3 eV), where the photon energy entering the sample is inadequate to activate the electrons in the polymer films. However, the photon energy is sufficient to excite the electrons from the valence band to the conduction band in the high photon energy area (> 3 eV); as a result, the charge carriers will increase. Additionally, this raises optical conductivity^82^. The reported effect of Al_2_O_3_ nanoparticles on the optical conductivity of PVC-based nanocomposites can be explained by the intricate charge transfers that occur between the PVC molecules and the Al_2_O_3_ filler. Due to their ability to fit the interstitial space between the polymer strands and hence increase optical conductivity, Al_2_O_3_ nanoparticles can form a segregated network when they are distributed in PVC polymer^83^.

The electrical conductivity $\:{\sigma\:}_{e}$ is calculated using the following formula^84^:

5 $$\:{\sigma\:}_{e}=\frac{2\lambda\:{\sigma\:}_{opt}\:}{\alpha\:}$$

Figure 10b. depicts the dependency of $\:{\sigma\:}_{e}$ on the photon energy h$\:\nu\:$ for PVCPVC/x(Al_2_O_3_) (x = 0, 0.01, 0.03, 0.07, 0.1, 0.2 wt%). It is evident that when the energy of the incident photon increases, the electric conductivity decreases. The reason for this is the increase in absorbance. While it increases if the Al_2_O_3_ content is raised. This is a result of the growth of charge carriers.

When it comes to optical communication, the dispersion of the refractive index spectrum is crucial to study^85^. Wemple and DiDomenico’s (W–D) single oscillator model is used to study the refractive index dispersion behavior in the energy region of hv < E_g_^86^. Two parameters are included in the single oscillator theory: oscillator energy (E_0_) and dispersion energy (E_d_). Whereas the E_0_ variable represents a mean energy band gap, the E_d_ value gauges the saverage interband optical transition strength. It is closely related to the charge distribution inside the unit cell and, consequently, to its chemical bond^87^. The following scientific equation establishes a relationship between photon energy (hν) and refractive index (n)^88^:

6 $$\:{\left({n}^{2}-1\right)}^{-1}=\frac{{E}_{0}}{{E}_{d}}-\left(\frac{1}{{E}_{0}{E}_{d}}\right){\left(h\nu\:\right)}^{2}$$

**Fig. 11:**
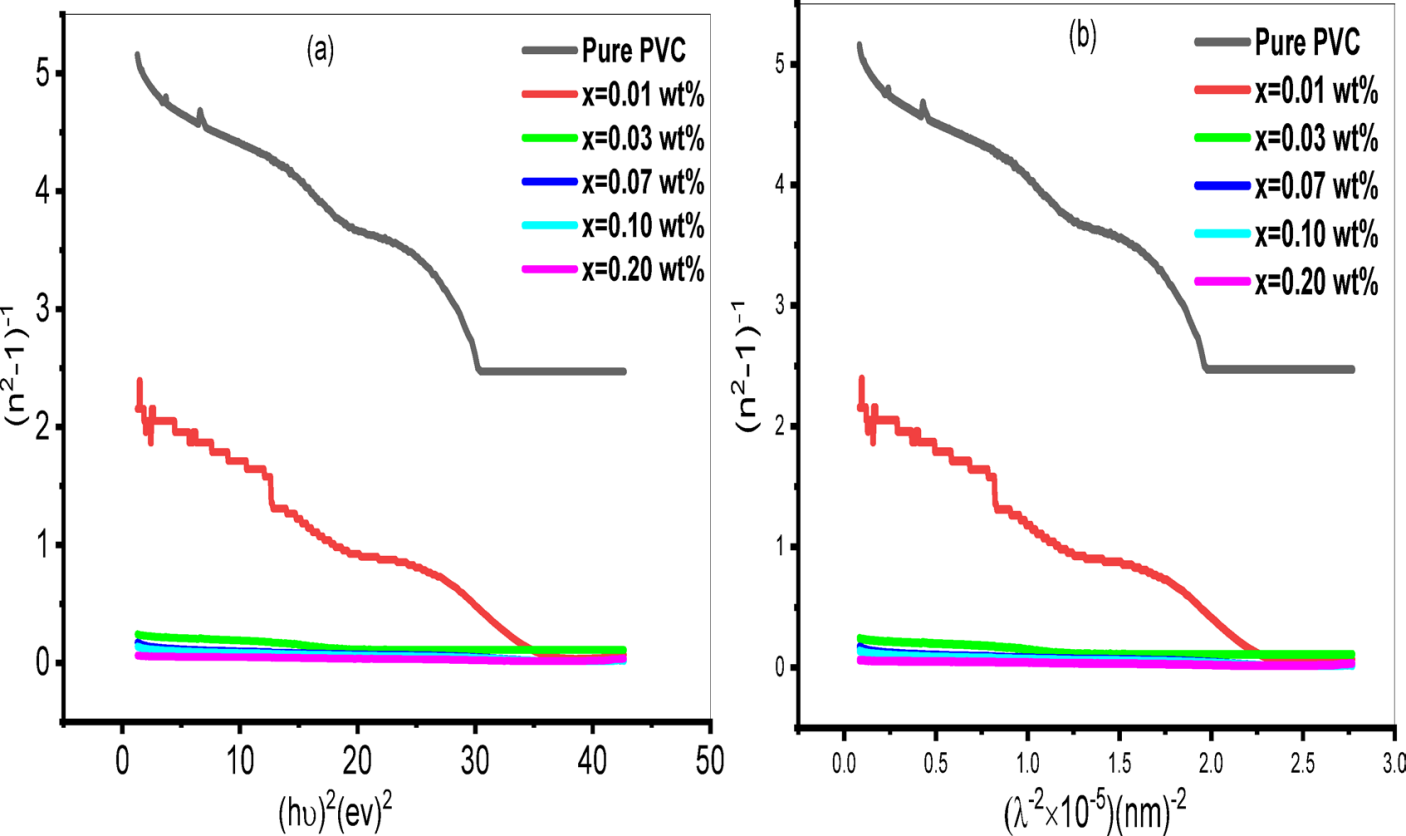
**(a)** Variation of (n^2^−1)^−1^ with photon energy for PVC/x (Al_2_O_3_) (x = 0, 0.01, 0.03, 0.07, 0.1, 0.2 wt%) nanocomposite **(b)** Variation of (n^2^−1)^−1^ with wavelength for PVC/x (Al_2_O_3_) (x = 0, 0.01, 0.03, 0.07, 0.1, 0.2 wt%) nanocomposite.

Based on the slope and intercept of the linear portion of the $\:{\left({n}^{2}-1\right)}^{-1}$ plot versus $\:{\left(h\nu\:\right)}^{2}$ close to the edge of absorption, as seen in Fig. 11a, the oscillation parameter values E_0_ and E_d_ are determined. Table 3 lists the dispersion parameters (E_0_ and E_d_) that are defined. The dispersion energy (E_d_) rises, whereas the oscillator energy (E_0_) value decreases as the concentration of Al_2_O_3_ nanoparticles increases, as Table 3 makes clear. This increase in E_d_ is due to the creation of a charge transfer complex between Al_2_O_3_ nanoparticles and PVC polymer. Increased concentration of Al_2_O_3_ nanoparticles in the PVC matrix causes the localized electronic states of the HOMO-LUMO gap to expand, improving the low energy transitions and reducing the values of E_0_^89^. The direct optical energy band gap (E_g_), which is determined using Tauc’s method, and the oscillator energy (E_0_) value shown in Table 3 have a similar filler dependence.

The oscillator variables are used to determine the optical spectra moments (M_− 1_ and M_− 3_), which quantify the light-material interacting mechanism^90^:

7 $$\:{M}_{-1}=\frac{\mathrm{E}\mathrm{d}\:\:}{\mathrm{E}0\:\:},\:\:\:\:{M}_{-3}=\frac{{M}_{-1}\:\:}{{E}_{0}^{2}\:\:}$$

Values of M_− 1_ and M_− 3_ after estimating are summarized in Table 3, it is discovered that the values of M_− 1_ and M_− 3_ rise as the Al_2_O_3_ level does. An additional optical parameter is the oscillator strength (f), which describes how the electron absorbs photons between its initial and ultimate state is calculated by^91^:

8 $$\mathrm{F}=\mathrm{E}_0 *\mathrm{E}_\mathrm{d}$$

and tabulated in Table 3. The next equation is used also to calculate my analyzed samples’ static refractive index or zero frequency refractive index, (n_0_)^92^:

9 $$\:{n}_{0}=\sqrt{1+\frac{{E}_{d}}{{E}_{0}}}$$

Therefore, the linear static refractive index (n_0_) values for each sample is used to estimate the static dielectric constant ($\:{\varepsilon\:}_{s}$) using the formula below^93^:

10 $$\:{\varepsilon\:}_{s}=\:{n}_{0}^{2}$$

The calculated values of static refractive index and static dielectric constant are summarized in Table 3. The findings showed that when the concentration of Al_2_O_3_ increases, so do the values of n_0_, $\:{\varepsilon\:}_{s}$, and f. These composites’ increased refractive index increases their potential for usage in a variety of applications, such as high refractive index lenses and reflecting coatings for solar cell systems.

**Table 3:** The optical dispersion and moments parameters of the PVC/x (Al_2_O_3_) (x = 0, 0.01, 0.03, 0.07, 0.1, 0.2 wt%) nanocomposite films.

Concentration (wt%)	E_0_ (oscillator energy) (eV)	E_d_ dispersion energy (eV)	M_− 1_ (eV)	M_− 3_ (eV)	F Oscillator strength (eV)^2^	*n*_0_ (static refractive index)	$\:{\varepsilon\:}_{s}\:(\:$static dielectric constant)
Pure PVC	3.95	0.72	0.18	0.01	2.84	1.08	1.16
X = 0.01	3.45	1.44	0.41	0.03	4.96	1.19	1.41
X = 0.03	3.32	12.63	3.80	0.34	41.93	2.18	4.75
X = 0.07	2.35	13.93	5.92	1.07	32.73	2.46	6.05
X = 0.1	2.05	15.54	7.58	1.8	31.85	2.70	7.29
X = 0.2	1.5	48.74	32.49	14.44	73.11	4	16

It is necessary and crucial to investigate nonlinear optical factors, including refractive index and susceptibility, for applications related to optical switching, photonics, and self-focusing. Whereas the material becomes polarized when exposed to an optical field, which leads to nonlinearity. For many applications, including capacitive communication systems, third-order nonlinear optical susceptibility, (χ^3^) and nonlinear refractive index (n_2_) are regarded as crucial nonlinear optical characteristics^94^. Using the acquired values of E_0_, E_d_, and n_0_, these nonlinear optical parameters are computed using the following equations^95^ :

11 $$\:{\chi}^{1}=\raisebox{1ex}{${E}_{d}$}\!\left/\:\!\raisebox{-1ex}{$4\pi\:{E}_{0}$}\right.$$

12 $$\:{\chi}^{3}=6.82\times\:{10}^{-15}{\left(\frac{{E}_{d}}{{E}_{0}}\right)}^{4}$$

13 $$\:{n}_{2}=\raisebox{1ex}{$12\pi\:{\chi}^{3}$}\!\left/\:\!\raisebox{-1ex}{${n}_{0}$}\right.$$

Table 4 displays the computed values of χ^1,^ χ^3^, and n_2_. It is evident that when the concentration of Al_2_O_3_ nanoparticles in the PVC matrix increased, so did the values of χ^1^, χ^3^, and n_2_. The presence of Al_2_O_3_ nanoparticles in the polymer matrix is connected to the high value of χ^3^^96^. It has to do with how the incident photon energy affects the refractive index^97^. High n_2_ values suggest that the PVC/Al_2_O_3_ composite samples under study would make intriguing nonlinear optical application possibilities.

**Table 4:** Non-linear optical parameter of PVC/x (Al_2_O_3_) (x = 0, 0.01, 0.03, 0.07, 0.1, 0.2 wt%) composite films.

Concentration (wt%)	linear optical susceptibility χ^1^	third-order nonlinear optical susceptibility χ^3^	nonlinear refractive index *n*_2_
Pure PVC	0.01	7.73$\:\times\:$10^−18^	2.68$\:\times\:$10^−16^
X = 0.01	0.03	2.06$\:\times\:$10^−16^	6.55$\:\times\:$10^−15^
X = 0.03	0.3	1.41$\:\times\:$10^−12^	2.43$\:\times\:$10^−11^
X = 0.07	0.4	4.5$\:\times\:$10^−12^	6.9$\:\times\:$10^−11^
X = 0.1	0.5	1.09$\:\times\:$10^−11^	1.52$\:\times\:$10^−10^
X = 0.2	1.19	3.48$\:\times\:$10^−10^	3.27$\:\times\:$10^−9^

The modified Wemple-Didomenico model is used for estimating a static optical refractive index ($\:{n}_{\infty\:}$) at an infinity wavelength^98^:

14 $$\:\frac{{{n}_{\infty\:}}^{2}-1}{{n}^{2}-1}=1-{\left(\frac{{\lambda\:}_{0}}{\lambda\:}\right)}^{2}$$

Where $\:{\lambda\:}_{0}$ is the interband oscillator’s average wavelength. Figure 11b. illustrates the association between $\:{\left({n}^{2}-1\right)}^{-1}\:$in contrast to $\:{\lambda\:}^{-2}$. The values of $\:{\lambda\:}_{0}$ and $\:{n}_{\infty\:}$ for the prepared samples are listed in Table 5. The values of $\:{\lambda\:}_{0}\:$and $\:{n}_{\infty\:}$ are found to increase with increased Al_2_O_3_ concentration; this might be attributed to an increase in packing density^99^.

The values of the lattice dielectric constant ($\:{\varepsilon\:}_{l}$) are determined using the relationship between ($\:{\varepsilon\:}^{{\prime\:}}$) and ($\:{\lambda\:}^{2}$) in the following way^100^:

15 $$\:{\varepsilon\:}^{{\prime\:}}{{=n}^{2}--{k}^{2}=\varepsilon\:}_{l}-\frac{{e}^{2}N}{4\pi\:{c}^{2}{{m}^{*}\varepsilon\:}_{0}}{\lambda\:}^{2}$$

16 $$\:{\varepsilon\:}^{{\prime\:}{\prime\:}}=2\mathrm{n}\mathrm{k}$$

**Fig. 12:**
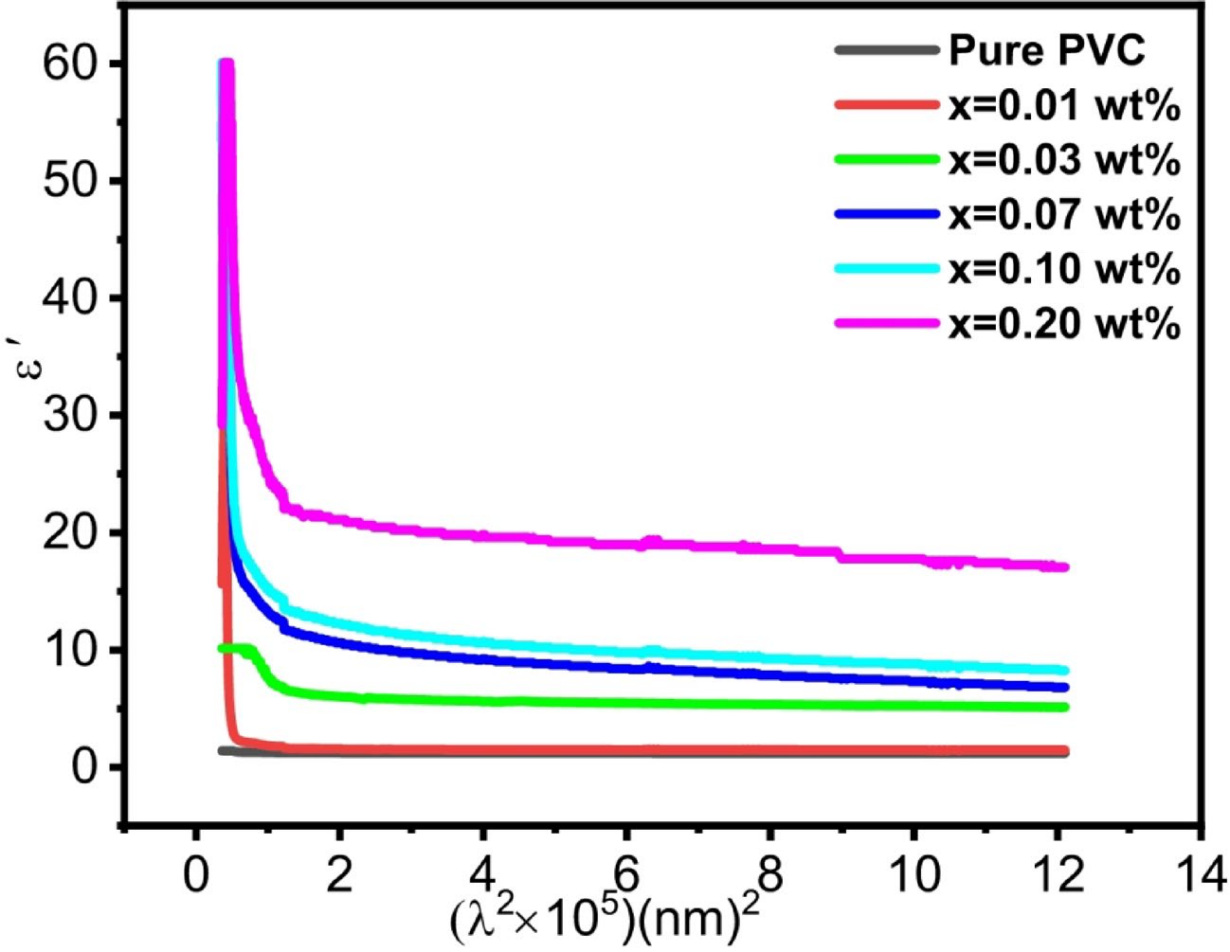
Variation of dielectric constant real part for PVC/x (Al_2_O_3_) (x = 0, 0.01, 0.03, 0.07, 0.1, 0.2 wt%) composite with wavelength.

Where e, c, $\:{\varepsilon\:}_{0}$, N/m* are electron charge, speed of light, permittivity of air, and free carrier concentration N to the effective mass $\:{\mathrm{m}}^{\mathrm{*}}$ ratio, respectively. For all samples, Fig. 12 shows how $\:{\varepsilon\:}^{{\prime\:}}$ depends on $\:{\lambda\:}^{2}$. Using the slope and intercept of Fig. 12, the values of (N/m*) and $\:{\varepsilon\:}_{l}$ are computed and given in Table 5. It is shown that when the content of Al_2_O_3_ increases, both N/$\:{m}^{*}$ and $\:{\varepsilon\:}_{l}\:$grew, which suggests an increase in free carriers. The findings demonstrated that the $\:{\varepsilon\:}_{l}$ values are greater than the $\:{\varepsilon\:}_{s}\:$ values. This discrepancy can be explained by both the rise in the concentration of free carriers and the process of polarization that takes place in the substance once light strikes it^101^.

The following formula is used to compute the plasma frequency ($\:{\omega\:}_{p}$) values of prepared samples using the values of (N/m*), and the results are reported in Table 5:

17 $$\:{\omega\:}_{p}={\left(\frac{{e}^{2}N}{{{{\varepsilon\:}_{l}m}^{*}\varepsilon\:}_{0}}\right)}^{0.5}$$

The elevated plasma frequency is ascribed to high levels of free carriers^100^.

**Table 5:** Optical refractive index ($\:{n}_{\infty\:}$) at an infinity wavelength, average interband oscillator wavelength ($\:{\mathsf{\lambda\:}}_{0}$), free carrier concentration N to the effective mass $\:{\mathrm{m}}^{\mathrm{*}}$ ratio (N/$\:{\mathrm{m}}^{\mathrm{*}}$), lattice dielectric constant $\:\left({{\upepsilon\:}}_{\mathrm{l}}\right)\:\:$ and the plasma resonance frequency $\:({{\upomega\:}}_{\mathrm{p}}$) of PVC/x (Al_2_O_3_) (x = 0, 0.01, 0.03, 0.07, 0.1, 0.2 wt%) composite films.

Concentration (wt%)	$\:{n}_{\infty\:}$	$\:{\lambda\:}_{0}$ $\:\left(nm\right)$	$\:{\varepsilon\:}_{l}$	(*N*/$\:{m}^{*}{10}^{57})$ $\:\left({\:{kg}^{-1}\:m}^{-3}\right)$	$\:{(\omega\:}_{p}\:\times\:{10}^{13})$ $\:\left({sec}^{-1}\right)$
Pure PVC	1.09	159.39	1.22	0.28	0.7
X = 0.01	1.2	187.66	1.54	0.86	1.2
X = 0.03	2.26	192.44	6.05	9.3	4.1
X = 0.07	2.67	283.48	10.88	40.57	8.5
X = 0.1	2.91	296.85	12.45	42.24	8.7
X = 0.2	4.18	305.55	21.43	43	8.9

**Fig. 13:**
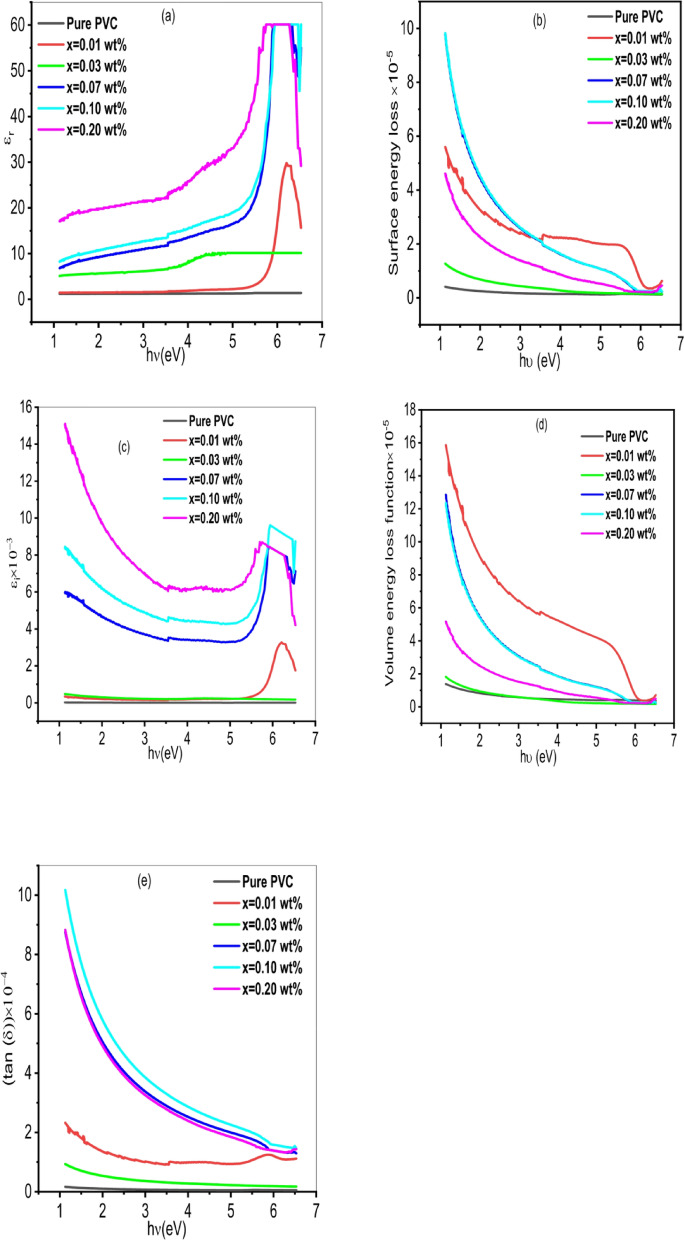
Dependence of **(a)** Real part of dielectric constant, **(b)** Surface energy loss **(c)** imaginary part of dielectric constant, **(d)** Volume energy loss, **(e)** dissipation factor for PVC/x(Al_2_O_3_) (x = 0, 0.01, 0.03, 0.07, 0.1, 0.2 wt%) composite on photon energy.

In optoelectronic and electronic applications, the estimation procedure for polymers relies on their dielectric constant properties. One of the key factors describing the performance of the polymer is the dielectric constant. The dielectric constant’s real portion ($\:{\varepsilon\:}_{r}$) indicates the dispersion process within the polymer and shows how the molecules’ polarizability affects the incident electromagnetic beam’s speed. The dielectric constant’s imaginary portion ($\:{\varepsilon\:}_{i}$) offers details about the incident electromagnetic energy loss in the medium or the material’s resistance to the photon incident beam. Additionally, the surface energy loss function (SELF) measures how the material’s surface can lose electron energies and the volume energy loss function (VELF) measures how quickly the generated fast electrons may lose energies in the bulk material. Figure 13. shows the changes in $\:{\varepsilon\:}_{r}$ SELF, $\:{\varepsilon\:}_{i}$ and VELF as a function of the incident photon energy for PVC/x(Al_2_O_3_) (x = 0, 0.01, 0.03, 0.07, 0.1, 0.2 wt%) composite. The real part of the optical dielectric constant $\:{(\varepsilon\:}_{r})\:$increases with increasing the incident photon energy. This is due to interactions between photons and electrons in the films that occur in this energy range^102^. The imaginary part of the optical dielectric constant $\:{(\varepsilon\:}_{i})\:$decreases with increasing incident photon energy. This decrease is due to the scattering behavior^103^. Both $\:{\varepsilon\:}_{r}$ and $\:{\varepsilon\:}_{i}\:$increase with the concentration of Al_2_O_3_ nanoparticles in the PVC matrix. Changes in the dielectric constant of various polymers, when filled with various additives, could be caused by many factors: (i) Modifications to the nanofillers’ electron/photon interaction and (ii) variations in the dipole motion of pure and loaded polymers^104^. In all energy photon ranges, it is found that the values of VELF and SELF follow the same trend and do not significantly differ^105^. Both $\:{\varepsilon\:}_{r}$ and VELF are greater than the equivalent $\:{\varepsilon\:}_{i}$ and SELF values in each sample. The rate at which a mechanical mode, like an oscillation, loses power in a dissipative system is known as the dissipation factor tan $\:\left(\delta\:\right)$, and it may be found using (tan $\:\left(\delta\:\right)$= $\:{\varepsilon\:}^{{\prime\:}{\prime\:}}$/$\:{\varepsilon\:}^{{\prime\:}}$)^106^. Figure 13e. shows how the optical dissipation factor tan $\:\left(\delta\:\right)$ for the samples under study depends on photon energy. It is found that the loss factors behaved similarly to the dielectric constants’ imaginary components. This lends credence to the theory that some photon-electron interactions occurred in the range of energies under study, and that these interactions are what led to the development of the dielectric spectra’s peaks^107,108^.

**Fig. 14:**
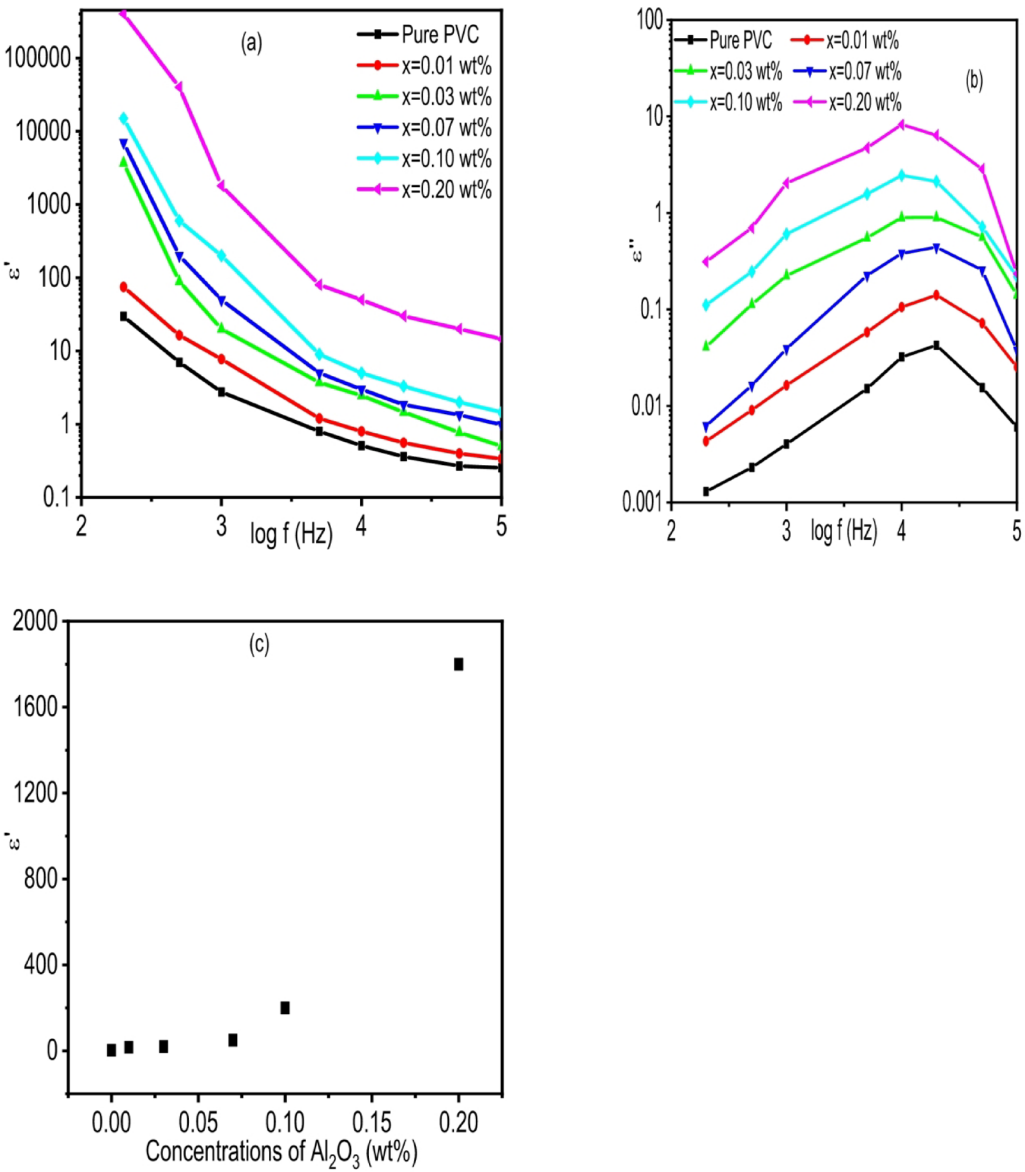
**(a)** Dependence of **(a)** Real and **(b)** imaginary part of dielectric constant for PVC/x (Al_2_O_3_) (x = 0, 0.01, 0.03, 0.07, 0.1, 0.2 wt%) composite on frequency. (c) variation of dielectric constant with Al_2_O_3_ concentrations.

Considering dielectric properties have significance for industrial applications like energy storage devices, researchers are very interested in studying them. With an external electric field, a material’s ability to polarize and hold onto charge is known as its dielectric constant^109,110^. Figure 14a and b show how the dielectric constant real and imaginary components (ε′, ε′′) depend on frequency (Log f), respectively. As depicted in Fig. 14a, the real component of the dielectric constant (ε′) shows a noticeable decrease with frequency across all samples, which aligns with the typical dielectric relaxation characteristics found in polymer-ceramic composites. At lower frequencies, ε′ reaches its maximum due to the prevalence of interfacial (Maxwell-Wagner-Sillars) polarization and the accumulation of space charge at the PVC/Al₂O₃ interfaces^111^. The ε′ value significantly increases with higher Al₂O₃ content, indicating that the ceramic filler creates more sites for polarization and enhances the ability to store charge^112^. The sample containing 0.20 wt% Al₂O₃ exhibits the highest ε′ values throughout the entire frequency spectrum, signifying a stronger interface polarization and an improved capacity to capture charge carriers, likely aided by the presence of agglomerated Al₂O₃ clusters noted in the SEM analysis^113^. As the frequency rises, ε′ declines sharply because dipolar and interfacial dipoles cannot keep up with the rapidly changing electric field, which results in a reduction of polarization mechanisms^114^. At elevated frequencies, ε′ values tend to stabilize at lower, nearly uniform levels, where only electronic polarization plays a role, suggesting minimal dispersion in this range^115^. The distinct frequency dispersion and trends in permittivity that depend on fillers indicate that the dielectric response is significantly influenced by space-charge and dipolar contributions at low frequencies, shifting to electronic polarization as the frequency increases^34^. The imaginary component of the dielectric permittivity (ε’’) offers essential insight into the mechanisms of energy dissipation within polymer materials exposed to alternating electric fields^116^. Figures (14b) shows how ε’’ varies with logarithmic frequency for pure PVC and PVC/xAl_2_O_3_ (x = 0, 0.01, 0.03, 0.07, 0.1, 0.2 wt%). In all samples, ε’’ displays a distinctive peak, signaling relaxation phenomena typically linked to interfacial polarization and dipolar reorientation^117^. At lower frequencies, the higher ε’’ values—particularly in samples with increased Al_2_O_3_ concentrations—indicate the prevalence of Maxwell–Wagner–Sillars (MWS) polarization, which results from charge accumulation at heterogeneous interfaces within the composite matrix^118^. A consistent increase in ε’’ is noted as the Al_2_O_3_ concentration rises from 0.01 wt% to 0.20 wt%. This rise in dielectric loss can be explained by the greater density of polarizable entities and the potential formation of conductive microdomains in the PVC matrix^119^. The Al_2_O_3_ is likely to enable localized charge transport and dipole–dipole interactions, thus enhancing the dielectric response^120^. While the frequency at which the ε’’ peak occurs remains largely unchanged, the amplitude of the peak increases with concentration, indicating more robust energy dissipation mechanisms^121^. This behavior aligns with the introduction of interfacial regions that encourage space charge polarization and boost the overall dielectric activity of the composite^12^. The reduction in ε’’ at higher frequencies reflects the incapacity of dipolar species to keep pace with the rapidly fluctuating electric field, leading to fewer dielectric losses^122^. This behavior is consistent with traditional dielectric relaxation theory, where the relaxation time (τ) determines the frequency at which maximum energy dissipation happens^123^. The broad and asymmetric characteristics of the ε’’ peaks indicate a range of relaxation times, which can be modeled using the Cole–Cole or Havriliak–Negami equations to derive quantitative parameters such as relaxation strength and shape factor^124^. These models are particularly effective for characterizing complex polymer systems that exhibit multiple overlapping relaxation processes^125^.As seen in Figure (14c), the concentration of Al_2_O_3_ was found to have a significant impact on the dielectric behavior of the composite system. As the Al_2_O_3_ content increased, the dielectric constant (ε_r_) increased gradually, ranging from 0.00 to 0.20 wt%. Interestingly, the increase in ε_r_ was nonlinear, with a sharp peak at 0.20 weight%, indicating that Al₂O₃ is essential for altering the polarization mechanisms in the matrix. Interfacial polarization effects and enhanced microstructural uniformity brought about by the ceramic filler may be responsible for this behavior^126^. Al_2_O_3_ is a promising additive for high-permittivity applications in electrical and energy storage devices, as the results show that even small additions can greatly improve the dielectric performance.

Recent progress in polymer nanocomposites has explored a variety of inorganic fillers—such as TiO₂, ZnO, La_2_O_3_, and Fe₃O₄ as shown in Table 6 integrated into PVC matrix with the aim of enhancing dielectric, magnetic, or UV shielding properties. However, these systems often require high filler concentrations (5–10 wt%), which can adversely affect optical clarity and flexibility, thus limiting their use in optoelectronic applications. In contrast, this research demonstrates that PVC/Al₂O₃ composites with very low loadings of Al₂O₃ 0.1–0.2 wt% exhibit improved structural, optical, and dielectric properties. The addition of Al₂O₃ nanoparticles increases the degree of crystallinity and diminishes lattice distortions, signifying enhanced structural regularity and stability within the PVC matrix. In terms of optical properties, the PVC/Al₂O₃ films present a significant decrease in the optical band gap, along with an increase in refractive index and absorption coefficient—attributes that are particularly advantageous for light-modulating and photonic applications. Additionally, Al₂O₃, known for its high-bandgap dielectric nature with excellent charge-trapping and thermal stability, enhances interfacial polarization and the dielectric constant without compromising the transparency or flexibility of the material. These combined properties make PVC/Al₂O₃ nanocomposites more effective and dependable candidates for optoelectronic and photonic device applications.

**Table 6:** Comparison of inorganic filler/PVC matrix systems.

Composites	Advantage	Ref
PVC/Al_2_O_3_	Enhanced each of crystallinity, visible absorption, and refractive index. *n* = 5 at λ = 400 nm, Eg = 3 eV,	Present work
PVC/ZnO	Improved dielectric properties, UV shielding, tuned morphology	^127^
PVC/ZnO	Improved insulation performance; aging behavior characterized	^128^
PVC plastisol + Ag@SiO2	Enhanced antimicrobial activity; improved mechanical/thermal properties	^129^
PVC + Cr _1.4_ Ca _0.6_ O_4_	Tunable optical, structural and thermal properties	^130^
PVC + modified ceramic particle	Mechanical reinforcement and thermal stability improvements	^131^
PVC-CUO/AL	Enhanced dielectric properties, Eg = 4.35 eV.	^19^
PVC/NiO	Enhanced AC conductivity, Eg = 3.8 eV.	^132^
PVC/ZnFe_2_O_4_	Enhanced dielectric properties, Eg = 3.88 eV.	^133^
PVC/La_2_O_3_	Eg = 5 eV.	^134^
PVA/Cs/TiO_2_	Eg = 4.23 eV.	^135^

## Conclusion

In this study, the structure, optical and dielectric properties of PVC were systematically optimized by adding Al₂O₃ nanoparticles with carefully controlled loading concentrations. XRD analysis revealed that the crystallinity of the polymer nanocomposite films increased as the concentration of Al₂O₃ nanoparticles increased. The incorporation of Al₂O₃ led to their agglomeration, a finding validated through XRD and SEM methods. SEM imaging confirmed that the Al₂O₃ nanoparticles presented a spherical shape at the nanoscale, displaying both isolated nanoparticles and some agglomeration within the polymer nanocomposite films. The UV–Vis spectra showed a significant redshift in the absorption edge along with increased absorbance in the visible range, reflecting substantial changes in electronic transitions and better light interaction properties. The direct optical band gap showed a reduction from 5.05 eV to 3 eV with rising Al₂O₃ nanoparticle concentration, suggesting that the polymer films exhibit semiconducting properties. The polymer nanocomposite films demonstrated a notably high refractive index of n (PVC/0.2 wt% Al₂O₃) = 4.8, approximately four times greater than the refractive index of pure PVC n (pure PVC) = 1.2. Nonlinear optical properties (χ¹, χ³, and n₂) were enhanced with entering Al_2_O_3_ into PVC polymer. The optical dielectric characteristics and optical conductivity of the films also increased with higher Al₂O₃ nanoparticle concentrations in the PVC matrix. The results indicated that low-cost PVC/xAl_2_O_3_(x = 0.1) nanocomposites could serve as a crucial element in advanced optoelectronic applications.

## Data Availability

The data that support the findings of this study are available from the corresponding author upon reasonable request.
